# Spatio‐Temporal Comparisons Between Microclimate Species Distribution Models and Mechanistic Models of Potential Surface Activity

**DOI:** 10.1002/ece3.71813

**Published:** 2025-07-16

**Authors:** Samuel FitzSimons Stickley, John A. Crawford, William E. Peterman, Jennifer M. Fraterrigo

**Affiliations:** ^1^ Department of Natural Resources and Environmental Sciences University of Illinois, Urbana‐Champaign Urbana Illinois USA; ^2^ National Great Rivers Research and Education Center East Alton Illinois USA; ^3^ School of Environment and Natural Resources The Ohio State University Columbus Ohio USA; ^4^ Department of Natural Resources and Environmental Sciences, Program in Ecology, Evolution, and Conservation Biology University of Illinois, Urbana‐Champaign Urbana Illinois USA

**Keywords:** climate change, ecological niche models, Great Smoky Mountains National Park, microhabitat, physiology, plethodontid salamanders

## Abstract

With projected decreases in biodiversity looming due to changing environmental conditions, it is important for conservation managers to have accurate predictions of species' distributions. Species distribution models (SDM) and mechanistic models that account for biophysical factors are important tools for predicting potential distributions for many species. Incorporating microclimate data into SDMs and mechanistic models has become an important step for developing biologically relevant models for organisms reliant on microclimatic regimes. However, there remains a need to compare and integrate the predictions from microclimate‐derived SDMs and mechanistic models at fine spatio‐temporal scales to improve predictive accuracy and quantify model uncertainty. We developed correlative SDMs and mechanistic models of potential resistance to surface activity for two salamanders using fine‐resolution (3 m) microclimate data. Models were produced for the Great Smoky Mountains National Park, USA during the 2010, 2030, and 2050 time periods. We determined the spatio‐temporal agreement between SDMs and mechanistic models to assess model uncertainty at varying spatial resolutions. We also modeled and assessed spatio‐temporal variability within potential activity corridors. We found that agreement between fine‐resolution microclimate SDMs and mechanistic models was generally poor and varied temporally, but model agreement increased and converged at varying coarser spatial resolutions. Furthermore, potential activity corridors spatio‐temporally varied and demonstrated increased habitat fragmentation under future projections. The findings from this study highlight a contradiction in which we may need to model species distributions with microclimate data at finer, more biologically meaningful resolutions, but model agreement between correlative and mechanistic approaches may be weakened at these fine scales. Researchers may therefore need to strike a balance between increasing spatial resolution and study extent when integrating model approaches. Further quantifying model uncertainty and identifying alternative methods for integrating SDMs and mechanistic models will be an important step towards accurately predicting species distributions under changing environmental conditions.

## Graphical abstract

Species distribution models (SDM) and mechanistic models that account for biophysical factors are important tools for predicting potential distributions for many species, but there remains a need to compare and integrate the predictions from microclimate‐derived SDMs and mechanistic models at fine spatio‐temporal scales to quantify and reduce model uncertainty. We assessed the spatio‐temporal agreement between microclimate SDMs and mechanistic models for terrestrial salamanders in Great Smoky Mountains National Park and found that the agreement between microclimate SDMs and mechanistic models was generally poor, varied across temporal periods, and was weakest at high spatial resolutions. The findings from this study highlight a contradiction in which we need to model species distributions with microclimate data at finer, biologically meaningful resolutions, but model agreement between correlative and mechanistic approaches may be weakened at these fine scales.

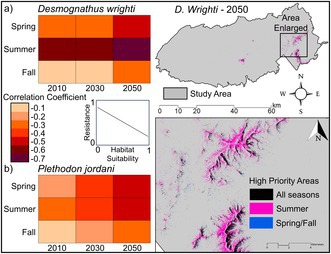

## 1 Introduction

Accurately delineating the distributions of species under changing environmental conditions has been a long‐standing goal for ecologists. Species distribution modeling has become the most common method for assessing species' current distributions and projected distributions under changing climate conditions (Elith et al. 2008; Lembrechts et al. 2018). However, species distribution models (SDMs) are typically based on climate data developed from free‐air temperature predictions at coarse spatial resolutions (Potter et al. 2013; Lenoir et al. 2017; Lembrechts et al. 2018), disregarding the important buffering effects that vegetation and biophysical interactions have on near‐surface climate conditions, that is, microclimate (Davis et al. 2018; De Frenne et al. 2019; Zellweger et al. 2019; Stickley and Fraterrigo 2021). In recent years, there have been novel efforts to develop SDMs with microclimate data that account for environmental conditions at scales appropriate to the biology of species reliant on varying microclimates (Lembrechts et al. 2018; Stark and Fridley 2022; Stickley and Fraterrigo 2023; Haesen et al. 2023). Advancements in the development of microclimate SDMs have been important for assessing species' distributions more accurately than approaches relying only on coarse‐resolution, free‐air climate data, that is, macroclimate conditions (Stark and Fridley 2022; Stickley and Fraterrigo 2023; Haesen et al. 2023). Nevertheless, there is a continued need to develop and integrate correlative SDMs and mechanistic modeling approaches that account for ecologically relevant microclimate conditions while simultaneously addressing the appropriate spatio‐temporal scales for the specific species being studied (Kearney and Porter 2009; Lembrechts et al. 2018).

SDMs are correlative, linking occurrence records of a species to environmental variables related to climate, topography, and habitat (Guisan and Zimmerman 2000; Guisan and Thuiller 2005; Elith et al. 2006). These methods are typically unable to capture fine‐scale variation in biological and physiological processes that affect the performance and fitness of organisms (Barton and Terblanche 2014; Evans et al. 2015). Alternatively, mechanistic models aim to account for factors that restrict the activity, movement, and redistribution of a species (Kearney and Porter 2009; Enriquez‐Urzelai et al. 2019). For example, biophysical models of evaporative water loss and desiccation have identified barriers to dispersal and movement for amphibians and reptiles (Riddell et al. 2017, 2018). Similarly, physiological models of metabolic activity have predicted potential range shifts due to variation in thermal conditions for a variety of organisms (Buckley et al. 2018). Mechanistic models developed with fine‐resolution microclimate datasets may be especially valuable for predicting the activity, movement, and other occupancy dynamics for species reliant on microclimatic regimes (Cushman 2006; Hoegh‐Guldberg et al. 2008; Kearney and Porter 2009; Hortal et al. 2010; Clusella‐Trullas et al. 2011). A variety of approaches are available for incorporating in situ or regionally downscaled microclimate estimates into mechanistic models (Peterman and Smlitsch 2013; Bennie et al. 2013; Hodgson et al. 2015; Ficetola et al. 2018). There are also approaches that combine globally available microclimate data and biophysical modeling (Tomlinson et al. 2019; Newman et al. 2022; Mi et al. 2022; Lewandrowski et al. 2024), for example using the R package “NicheMapR” (Kearney and Porter 2016; Kearney and Porter 2019; Kearney et al. 2021). Despite the value of combining mechanistic approaches with microclimate data for modeling species' distributions and activity patterns, most mechanistic models still rely on coarse‐resolution datasets which may not be useful for certain species or analyses.

Both mechanistic models and SDMs can provide relevant information about potential species' distributions and range limitations, which are important for conservationists to understand given that climate change is expected to drive future losses of biodiversity (Thomas et al. 2004; Maclean and Wilson 2011; Urban 2015). However, there have been limited efforts to compare mechanistic models and SDMs, even with some increasing efforts to integrate these modeling approaches over the past decade (see Tourinho and Vale 2023). Comparing and integrating these different approaches continues to be an important step in improving accuracy and quantifying and reducing model uncertainty. A streamlined, yet typically overlooked, approach to model comparison and integration is to develop SDMs and mechanistic models with identical fine‐scale microclimate inputs and then assess the spatio‐temporal agreement of predictions and how agreement varies with spatio‐temporal scale. Model agreement could bolster confidence in distributional predictions, while model disagreement might signal important complexities among modeling efforts (Hijmans and Graham 2006; Kearney and Porter 2009). For example, model disagreement might indicate differences in the dominant species‐environment relationships that underlie the predictions of each model, and their relative scale dependence (Fraterrigo et al. 2014; Fournier et al. 2017). Overall, these approaches can help improve predictions and quantify and reduce uncertainty in models of species' distributions, thereby assisting land managers tasked with protecting microhabitat for vulnerable species reliant on microclimatic conditions (Schuwirth et al. 2019).

In this study, we used fine‐resolution (3 m) maps of microclimate temperature (Stickley and Fraterrigo 2021, 2023) with physiological models produced from salamander body size measurements (Peterman et al. 2016; Kessler et al. 2024) to compare correlative SDMs and mechanistic models for two plethodontid species in Great Smoky Mountains National Park (GSMNP). Plethodontid salamanders are vulnerable to climate change (Milanovich et al. 2010) because these small, lungless, ectothermic species rely on microclimate conditions to exchange gases (cutaneous respiration) across their semi‐permeable skin (Petranka 1998) and increases in temperature can affect their energetic demands and overall fitness (Sears 2005; DuRant et al. 2007). Additionally, plethodontids have limited surface‐active periods and dispersal distances, which further emphasizes their reliance on microclimatic conditions (Feder 1983; Lutterschmidt and Hutchison 1997; Bernardo and Spotila 2006; Connette et al. 2015). We developed fine‐resolution, microclimate SDMs and mechanistic models during periods of increased salamander surface activity (March–November) under current and projected (2030 and 2050) climate conditions. Mechanistic models were derived by integrating salamander standard metabolic rate (SMR), which accounts for species‐specific body mass clines, with vapor pressure deficit (VPD) to target areas of land that may allow for increased salamander surface activity with lower energetic costs of maintenance. We compared model predictions at different spatial and temporal scales to characterize spatio‐temporal agreement and convergence between correlative SDMs and mechanistic models of potential resistance to surface activity. Lastly, we assessed the spatio‐temporal variability in potential activity corridors between locations of highly suitable microclimate habitat.

## 2 Materials and Methods

### 2.1 Study Area

**FIGURE 1:**
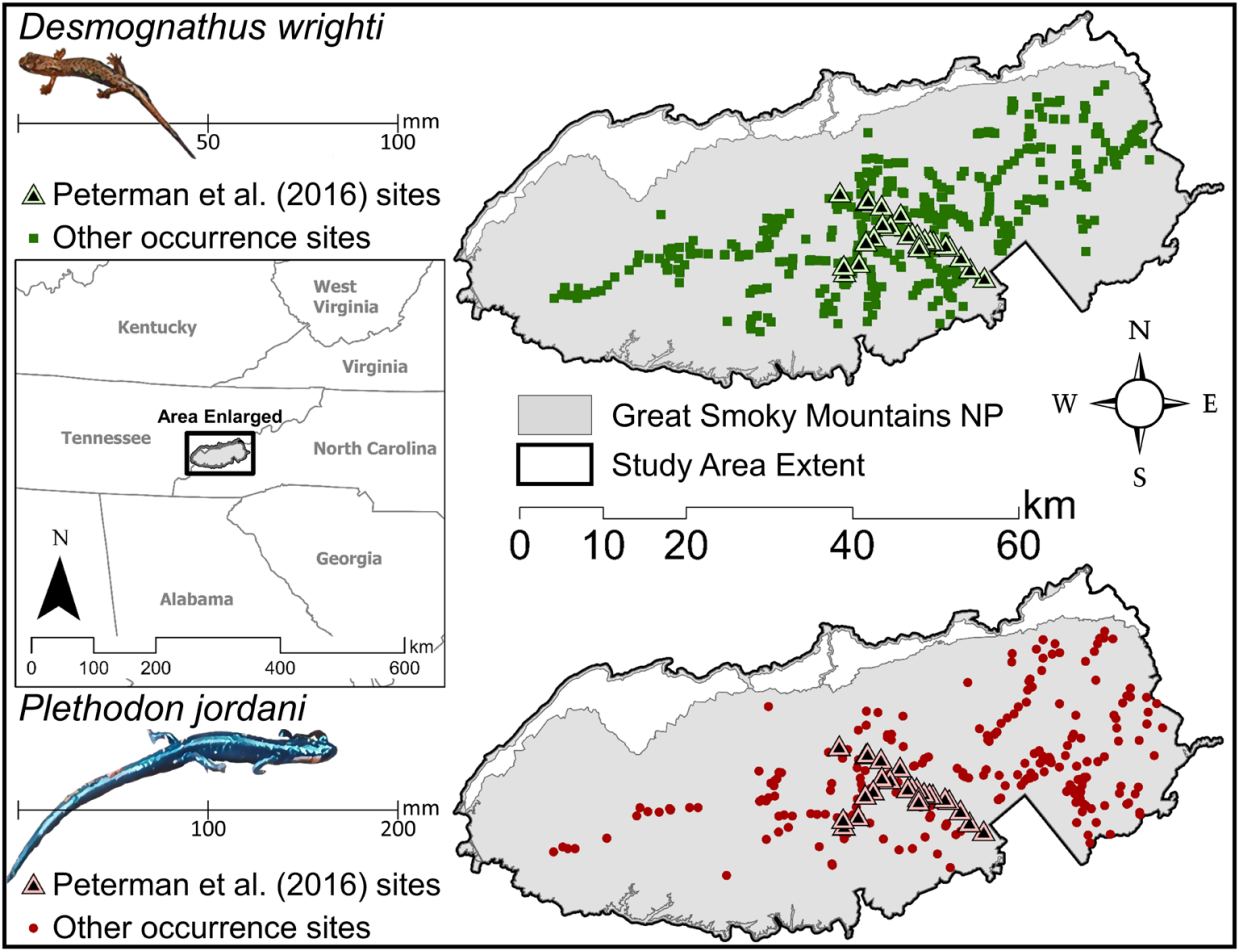
The location of Great Smoky Mountains National Park (GSMNP) and study area on the border of North Carolina and Tennessee in the southern Appalachian Mountains. Species occurrence locations for *Desmognathus wrighti* (top) and *Plethodon jordani* (bottom) include those collected from Peterman et al. (2016), symbolized as triangles. Species adult lengths (mm) are also depicted.

**FIGURE 2:**
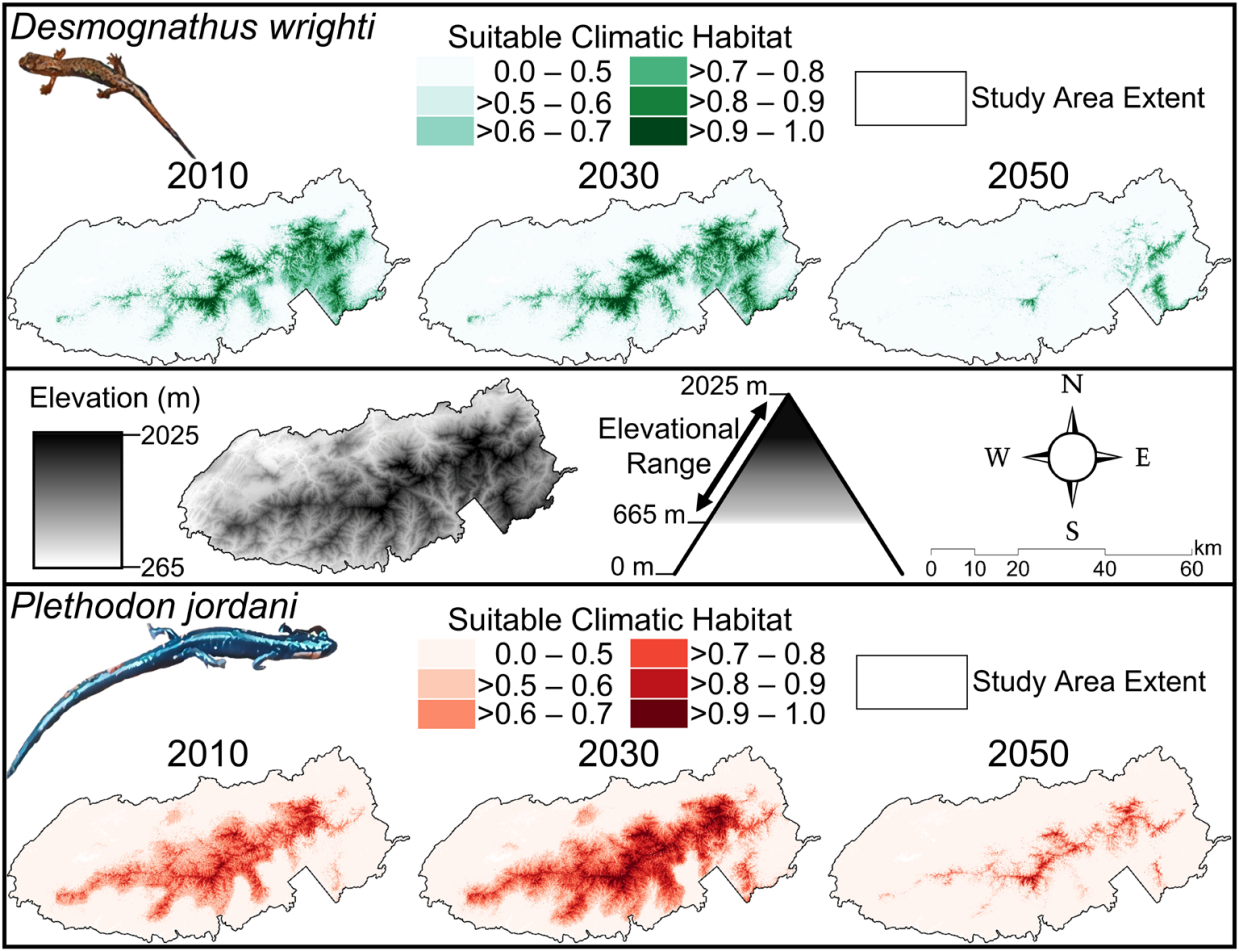
Microclimate species distribution models for *Desmognathus wrighti* (top) and *Plethodon jordani* (bottom). The probability of suitable climatic habitat is displayed for the 2010 (i.e., 2006–2010 average), 2030, and 2050 time periods. An elevation map of Great Smoky Mountains National Park is also shown (middle) with the typical elevational range for both species.

The study area for this project includes GSMNP with a 150 m buffer around the park boundary (Figure 1). Great Smoky Mountains National Park, located on the border of North Carolina and Tennessee, is one of the most visited National Parks in the United States. This region of the Southern Appalachian Mountains has elevated levels of biodiversity, including some of the highest levels of plethodontid salamander richness (Kozak and Wiens 2010; Gifford and Kozak 2012). Elevation in the Park ranges from 265 to 2025 m and the complex terrain, made up of mostly deciduous forests (Whittaker et al. 2007), provides the microclimatic regimes needed for salamanders to thrive (Figure 2; Petranka 1998).

### 2.2 Study Species

We focused on two fully terrestrial plethodontid salamander species with contrasting body sizes (Figure 1). The Pygmy Salamander ( *Desmognathus wrighti* ) is one of the smallest species of plethodontids in the region, ranging from about 13.6 to 33.0 mm in snout‐vent length (SVL) within GSMNP (Kessler et al. 2024) and rarely surpassing 50 mm SVL anywhere in their geographic range (Crespi et al. 2010). The Red‐cheeked or Jordan's Salamander ( *Plethodon jordani* ) is one of the larger plethodontid species in the region, ranging from approximately 27.4 to 74.0 mm in SVL at an adult stage (Peterman et al. 2016). Both species are widely distributed throughout GSMNP and are higher elevation specialists typically occurring from 665 to 2025 m (Figure 2; Dodd 2004; Peterman et al. 2016; Hocking et al. 2021).

### 2.3 Microclimate SDMs

We used ESRI ArcGIS Pro v. 3.1‐3.3 (2024) and R statistical software v. 4.1.2‐4.2.1 (R Core Team 2024) for all spatial analyses, and R statistical software v. 4.1.2‐4.2.1 for all statistical modeling. To develop correlative SDMs that capture relevant microclimate conditions, we used 11 bioclimatic variables as input data layers, calculated by Stickley and Fraterrigo (2023) from monthly minimum and maximum microclimate datasets at a 3 m spatial resolution (Stickley and Fraterrigo 2021; Table S1). The 11 chosen bioclimatic variables have been documented as biologically relevant for plethodontid salamanders (Rissler and Apodaca 2007; Milanovich et al. 2010). All climate variables were modeled during the growing season months (March–November) from 2006 to 2010 and used to estimate seasonal averages for spring (March–May), summer (June–August), and fall (September–November). Temperature averages from 2006 to 2010 were used to represent the 2010 time period. For future climate predictions, we used temperature models at a 3 m spatial resolution for 2030 and 2050 developed by Stickley and Fraterrigo (2023), which integrated the microclimate temperature datasets with future predictions from National Aeronautics and Space Administration (NASA) Earth Exchange Downscaled Climate Projections dataset (Thrasher et al. 2013; NASA NEX‐DCP30). All future climate projections were based on an ensemble model produced with Representative Concentration Pathway (RCP) 8.5. This emissions scenario has been recommended for future projections through the year 2050 because current trends have been in close alignment (Schwalm et al. 2020). See the Supporting Information and Stickley and Fraterrigo (2021, 2023) for additional modeling details.

To create a database of species' occurrence locations, we compiled historical occurrence data for our study species from Peterman et al. (2016), Milanovich et al. (2010), the Illinois Natural History Survey (https://herpetology.inhs.illinois.edu/databases/), the Smithsonian National Museum of Natural History (https://collections.nmnh.si.edu/search/), and the Hands on the Land Network (https://handsontheland.org/). Occurrence locations collected before 1990 were removed to retain data within 20 years of the initial time periods being modeled. After compilation, presence locations included 294 unique points for *D. wright* and 465 unique points for *P. jordani* (Figure 1).

Bioclimatic layers and occurrence data were used to develop correlative SDMs for both study species. To limit model uncertainty, we used an ensemble approach with three different SDM algorithms. First, we produced a maximum entropy (MaxEnt; Elith et al. 2011) model by following Hijmans and Elith (2017) with the “dismo” package. We also used the “SDMtune” package (Vignali et al. 2022) for model parameterization. We assessed MaxEnt models using *AICc* to choose the best model features and optimized regularization values by comparing all feature combinations and regularization values between 0 and 20 by increments of 0.1 (Table S2). We next used the “Biomod2” package (Thuiller et al. 2024) to develop SDMs with 10 separate algorithms using default settings. We chose the two top performing algorithms based on a comparison of the true skills statistic (TSS) for the 2010 SDM models; artificial neural network (ANN) and generalized boosted models (GBM) were selected for both species. We then calculated the ensemble model using the three model outputs (see Supporting Information).

For every SDM, we used area under the receiver‐operating characteristic curve (AUC) to assess model fit and we considered models with AUC ≥ 0.7 to be valid (Phillips and Dudik 2008). We evaluated model goodness‐of‐fit using *k*‐fold cross validation to ensure the use of all data points while reducing variance. Because the Continuous Boyce Index (CBI) has been shown to be a good metric for assessing model fit with presence‐only data, we also calculated CBI with a moving window size of 100 focals and the “spearman” correlation method for all of our final models using the ecospat package in R (Boyce et al. 2002; Di Cola et al. 2016). The SDMs produced for the 2010 time period were used to predict future suitable habitat with the bioclimate estimates for 2030 and 2050. We considered areas with a probability of suitable habitat ≥ 0.7 as highly suitable microclimate habitat (Qasim et al. 2024). Overall, we produced 18 microclimate SDMs (2 species × 3 time periods × 3 model algorithms) with six final ensemble model predictions (2 species × 3 time periods; see Supporting Information).

### 2.4 Microclimate Mechanistic Models

For salamanders, a low SMR means lower energetic costs of maintenance, which may help limit ecological trade‐offs that negatively affect overall fitness (Sears 2005; DuRant et al. 2007). Areas of the landscape that provide quality microclimate conditions (i.e., lower temperatures, increased moisture, etc.) help minimize physiological stress on salamanders (Cushman 2006; Kearney and Porter 2009). Vapor pressure deficit, which is the difference between the level of moisture in the air and the level of moisture that the air could hold at saturation, has also been shown to be a good indicator for salamander surface activity. Because warmer air increases the magnitude of drying, high VPDs may limit dispersal and foraging activities of salamanders (Riddell and Sears 2015; Riddell et al. 2017). Therefore, areas of the landscape with low VPDs and low SMRs may provide suitable habitat for surface activity with lower energetic demands on salamanders. To develop current and future mechanistic models of potential surface activity, we integrated mapped outputs of VPD and species‐specific SMR (Table S3).

We used monthly mapped outputs of current and future SMR (volume of oxygen consumption, VO_2_ μL g^−1^ h^−1^) from Stickley (2021). These SMR maps were developed for *D. wrighti* and *P. jordani* using the equations of Feder (1976, 1983), which predicted SMR for temperate plethodontid species as a function of temperature and body mass. The same microclimate temperature dataset used for the microclimate SDMs (Stickley and Fraterrigo 2021, 2023) was used for the temperature input of the SMR equations. Salamander body mass estimates, collected in situ by Peterman et al. (2016), were used to develop species‐specific predictive relationships between salamander body mass and elevation. See the Supporting Information for further information on salamander body mass and SMR modeling.

For VPD (kPa) estimates, we used minimum values because plethodontid salamanders are mostly surface active at night and increase surface and foraging activity under low VPD conditions (Riddell and Sears 2015; Gade et al. 2020). To model minimum VPD, we used the same microclimate temperature models from Stickley and Fraterrigo (2021, 2023) and specific humidity estimates from the Multivariate Adaptive Constructed Analogs (MACA) dataset (Abatzoglou and Brown 2012). We followed similar methodologies to Allen et al. (1998), using an ensemble model of specific humidity for future projections with the same RCP 8.5 emissions scenario used for our SDMs (see Supporting Information).

To produce the final mechanistic models, we rescaled the SMR and VPD raster outputs between 0.0 and 1.0 for each season and time period, then summed the rescaled values from both layers to assess areas where lower energetic costs of maintenance (low SMR) and lower resistance to surface activity (low VPD) occur across the park. The resulting layers were multiplied by 100 to develop resistance raster layers for least‐cost path modeling. Low values indicate areas with lower resistance to potential surface activity, and high values indicate areas with higher resistance to potential surface activity. We also delineated high priority areas (HPA) where low SMR (≤ median) and low VPD (≤ median) spatially coincided within the species' elevational ranges to identify areas of high value.

### 2.5 Comparing Microclimate SDMs and Mechanistic Models

We assessed the agreement between correlative SDMs and mechanistic models by calculating correlation coefficients between the mapped outputs, derived from the identical fine‐resolution microclimate data. Because low values from the mechanistic models indicate increased potential for surface activity and high values from SDMs indicate higher habitat suitability, negative correlations between the models demonstrated model agreement and positive values indicated model disagreement. We also tested correlations individually for the fine‐scale (3 m) SDMs with the SMR and VPD models separately, with negative correlations also representing model agreement. Calculations at such fine spatial resolutions are computationally expensive, so we randomly selected 100,000 points (“Generate Random Points” tool; ESRI 2024) across the elevational range of both study species (665–2025 m), then we extracted values representing the probability of suitable habitat from microclimate SDMs and values from our mechanistic outputs. We calculated correlation coefficients for each season and year of the models within each species elevation range and again within only the highly suitable microclimate habitat (≥ 0.7 probability) based on our microclimate SDMs. All variables were tested for spatial autocorrelation and statistical assumptions for correlation analysis. Although we did not find any evidence of spatial autocorrelation, some variables displayed non‐normal distributions or demonstrated non‐linear or dependent relationships. Therefore, we used the nonparametric Spearman's correlation coefficient (Spearman's *ρ*) for assessing agreement among correlative SDMs and mechanistic models. To compare model agreement across differing spatial resolutions, we first aggregated the microclimate SDMs and mechanistic models from 3 to 30 m. We then produced outputs with resolutions from 50 to 1000 m using a stepwise aggregation at every 50 m increment. We followed the same steps to calculate the correlation coefficient for each season, year, and spatial resolution, but we removed duplicate points (i.e., points that fell within a raster grid more than one time).

### 2.6 Activity Corridors

To model potential activity corridors for each season, time period, and species, we used least‐cost path (LCP) analysis with the Linkage Mapper v. 3.1.0. Toolbox (McRae and Kavanagh 2011). The microclimate mechanistic models were used as the input resistance rasters (i.e., resistance to potential salamander surface activity). To develop “core areas,” we used our microclimate SDMs and the “Region Group” tool from ArcGIS Pro (ESRI 2024) to group all ≥ 1 km^2^ contiguous areas of highly suitable microclimate habitat. We then modeled LCPs between the core areas across the resistance rasters following methodologies for the “Linkage Pathways Tool” (McRae and Kavanagh 2011). We buffered the LCPs by 300 m to estimate corridors, because plethodontid species have been shown to typically move less than 25 m from their habitat with the potential for up to 300 m movements (Welsh and Lind 1992; Marsh et al. 2004; Connette and Semlitsch 2013; NatureServe 2024). To determine how fragmentation patterns of suitable habitat within potential activity corridors changed across time periods, we clipped the highly suitable microclimate habitat (≥ 0.7 probability) within the activity corridors and calculated three landscape metrics, including the mean Euclidean nearest‐neighbor distance (ENN) in which higher values indicate increased distances between habitat fragments (Hargis et al. 1998), the patch density (PD) in which higher values suggest increased habitat fragmentation (McGarigal and Marks 1995), and the perimeter‐to‐area ratio (PA) in which higher values indicate increasingly complex habitat shapes and increased edge habitat (see Supporting Information for details).

## 3 Results

### 3.1 Microclimate SDMs

All the final ensemble microclimate SDMs were found to have good model fit based on the AUC and CBI metrics (Table 1, Figure 2, Table S2). The average AUC values were 0.84 and 0.83 for *D. wrighti* (2010 = 0.84, 2030 = 0.85, 2050 = 0.82) and *P. jordani* (2010 = 0.84, 2030 = 0.84, 2050 = 0.82), respectively. The average CBI values were 0.96 and 0.98 for *D. wrighti* (2010 = 0.97, 2030 = 0.98, 2050 = 0.94) and *P. jordani* (2010 = 0.99, 2030 = 0.98, 2050 = 0.97), respectively. All input SDMs to the ensemble models indicated moderate to good levels of accuracy, ranging from 0.79 to 0.86 AUC depending on the model (MaxEnt, ANN, GBM) and time period (Table S2, Figures S1–S3). From 2010 to 2050, the area of highly suitable microclimate habitat (≥ 0.7 probability of suitable habitat) decreased by 86% (area of suitable habitat in 2010 = 302.2 km^2^, 2050 = 42.2 km^2^) for *D. wrighti* and 76% (area of suitable habitat in 2010 = 203.6 km^2^, 2050 = 49.5 km^2^) for *P. jordani* (Table 1, Figure 2). For both species, we observed gains in highly suitable microclimate habitat between the 2010 and 2030 time period with considerable decreases by 2050 (Table 1, Figure 2).

**TABLE 1:** Results for microclimate species distribution models (SDM), correlations between SDMs and mechanistic models, and landscape metrics for *D. wrighti* and *P. jordani* for each season and study period. Area under the curve (AUC), Continuous Boyce Index (CBI), and the total area of highly suitable habitat (i.e., ≥ 0.7 probability) are shown for final microclimate SDMs. Correlation coefficients (Spearman's *ρ*) between correlative SDMs and mechanistic model outputs are shown for the entire species' ranges and within areas of highly suitable habitat. Landscape metrics include mean Euclidean nearest‐neighbor (ENN) with standard deviation, patch density (PD), perimeter‐area ratio (PA), and distance of least‐cost path (LCP) corridors.

Time period	*Desmognathus wrighti*			*Plethodon jordani*		
	2010	2030	2050	2010	2030	2050
Microclimate species distribution models						
AUC value	0.84	0.85	0.82	0.84	0.84	0.82
CBI value	0.97	0.98	0.94	0.99	0.98	0.97
Area of highly suitable habitat (km^2^)	302.18	312.91	42.18	203.63	445.31	49.50
Correlation Coefficients for SDMs and mechanistic models						
Spring	−0.30 (< 0.01)	−0.35 (0.05)	−0.51 (0.06)	−0.29 (−0.22)	−0.44 (−0.22)	−0.52 (< 0.01)
Summer	−0.63 (−0.13)	−0.66 (−0.12)	−0.77 (−0.02)	−0.33 (−0.22)	−0.45 (−0.37)	−0.53 (0.08)
Fall	−0.12 (< 0.01)	−0.17 (0.05)	−0.33 (0.05)	−0.15 (−0.17)	−0.29 (−0.07)	−0.34 (0.01)
Euclidean nearest neighbor (m)						
Spring	7.35 (6.99)	7.33 (7.08)	11.48 (15.90)	7.07 (6.45)	8.70 (10.86)	8.99 (10.71)
Summer	7.42 (7.15)	7.31 (6.93)	11.58 (16.13)	7.11 (6.50)	8.76 (11.76)	8.88 (10.12)
Fall	7.80 (8.48)	7.55 (7.70)	11.16 (15.48)	7.39 (6.91)	8.56 (11.17)	9.02 (10.77)
Patch density (patch per hectare)						
Spring	41.39	41.73	77.59	30.69	39.28	59.74
Summer	42.37	41.17	79.07	31.21	39.76	60.12
Fall	40.61	41.43	77.80	35.52	37.39	60.98
Perimeter‐area ratio (m)						
Spring	0.32	0.32	0.41	0.28	0.31	0.36
Summer	0.33	0.32	0.42	0.28	0.31	0.36
Fall	0.32	0.32	0.42	0.30	0.30	0.36
LCP distance between highly suitable habitat (km)						
Spring	2.48	3.20	6.65	2.35	1.62	4.79
Summer	2.50	3.23	6.73	2.36	1.64	4.74
Fall	2.55	3.37	6.75	2.36	1.65	4.85

### 3.2 Microclimate Mechanistic Models

Standard metabolic rate (VO_2_ μL g^−1^ h^−1^) increased by an average of 24.3% for *D. wrighti* and 14.6% for *P. jordani* between 2010 and 2050 (Table S3). Seasonally, SMR rates were 88.8% higher during summer periods for *D. wrighti* and 113.3% higher for *P. jordani* , compared to average values for spring and fall (Table S3). Vapor pressure deficit (kPa) was highest during spring with an average VPD of 0.95 kPa, compared to 0.83 kPa and 0.72 kPa in summer and fall, respectively (Table S3). On average, estimated VPD increased by 26.2% in spring, 47.8% in summer, and 37.7% in fall between 2010 and 2050. Resistance to potential surface activity increased by an average of 18.2% for *D. wrighti* and 17.9% for *P. jordani* between 2010 and 2050 (Table S4, Figures S4 and S5). Seasonally, resistance to potential surface activity was much higher in the summer period and increased by an average of 44.7% for *D. wrighti* and 54.9% for *P. jordani* from spring to summer. By 2050, seasonal increases in resistance to potential surface activity from spring to summer were over 8% higher for *D. wrighti* and over 7% higher for *P. jordani* compared to 2010 levels.

### 3.3 Spatio‐Temporal Model Comparison

**FIGURE 3:**
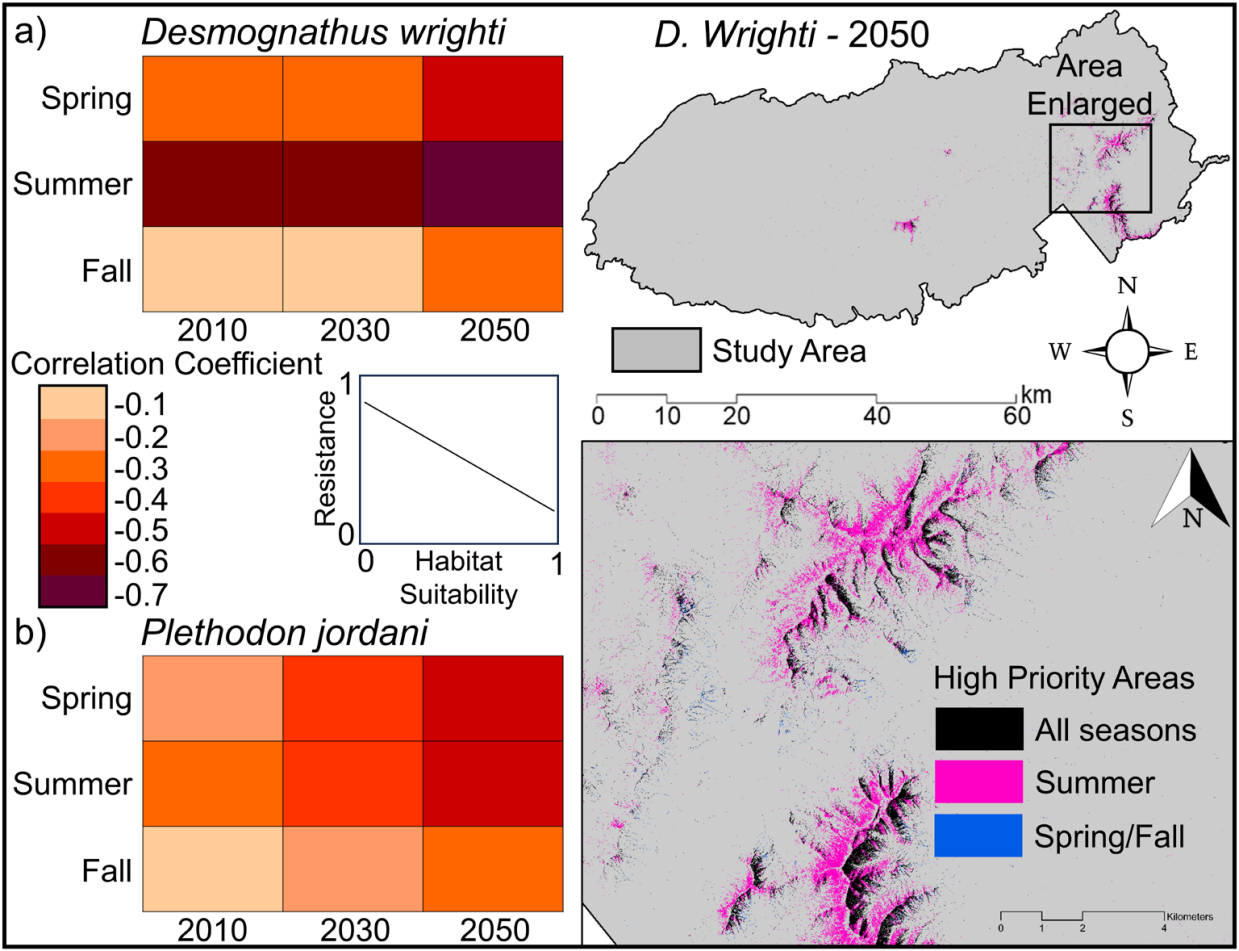
Spatio‐temporal correlation matrices for (a) *Desmognathus wrighti* and (b) *Plethodon jordani* . Correlation coefficients (Spearman's *ρ*) indicate the level of agreement between microclimate species distribution models and resistance to surface activity from microclimate mechanistic models across the study extent at a 3 m resolution. Also displayed is an example map of high priority areas (i.e., areas where low SMR and low VPD co‐occur) for *D. wrighti* projections in 2050 with high priority areas across all seasons (black) or only in summer (pink) or spring/fall (blue).

Our analysis indicates low to moderate agreement between microclimate SDMs and microclimate mechanistic models (Table 1, Figure 3). The average correlation coefficient for both species was −0.40 ( *D. wrighti* average = −0.43, *P. jordani* average = −0.37), ranging from −0.12 to −0.77 (Table 1). Although correlation coefficients were typically low to moderate, they were consistently negative, indicating that resistance to potential surface activity decreases as microclimate suitable habitat increases (Table 1, Figure 3). Model agreement was consistently highest during the summer ( *D. wrighti* average = −0.68, *P. jordani* average = −0.44) and lowest during the fall ( *D. wrighti* average = −0.21, *P. jordani* average = −0.26) for all time periods (Table 1, Figure 3). Within areas of highly suitable microclimate habitat, model agreement was consistently weak for both species, with correlation coefficients ranging from −0.13 to 0.06 for *D. wrighti* and −0.37 to 0.08 for *P. jordani*, respectively (Table 1, Figure S6).

We also found low to moderate agreement between our fine‐resolution, microclimate SDMs and the individual input variables (SMR and VPD) to the mechanistic model (Figure S7). For SMR, correlation patterns were similar to those of the full mechanistic model, with higher correlations in summer and during the 2050 study period, especially for *D. wrighti* . For VPD, moderate correlations were found during spring, with lower correlations in summer and fall (Figure S7).

**FIGURE 4:**
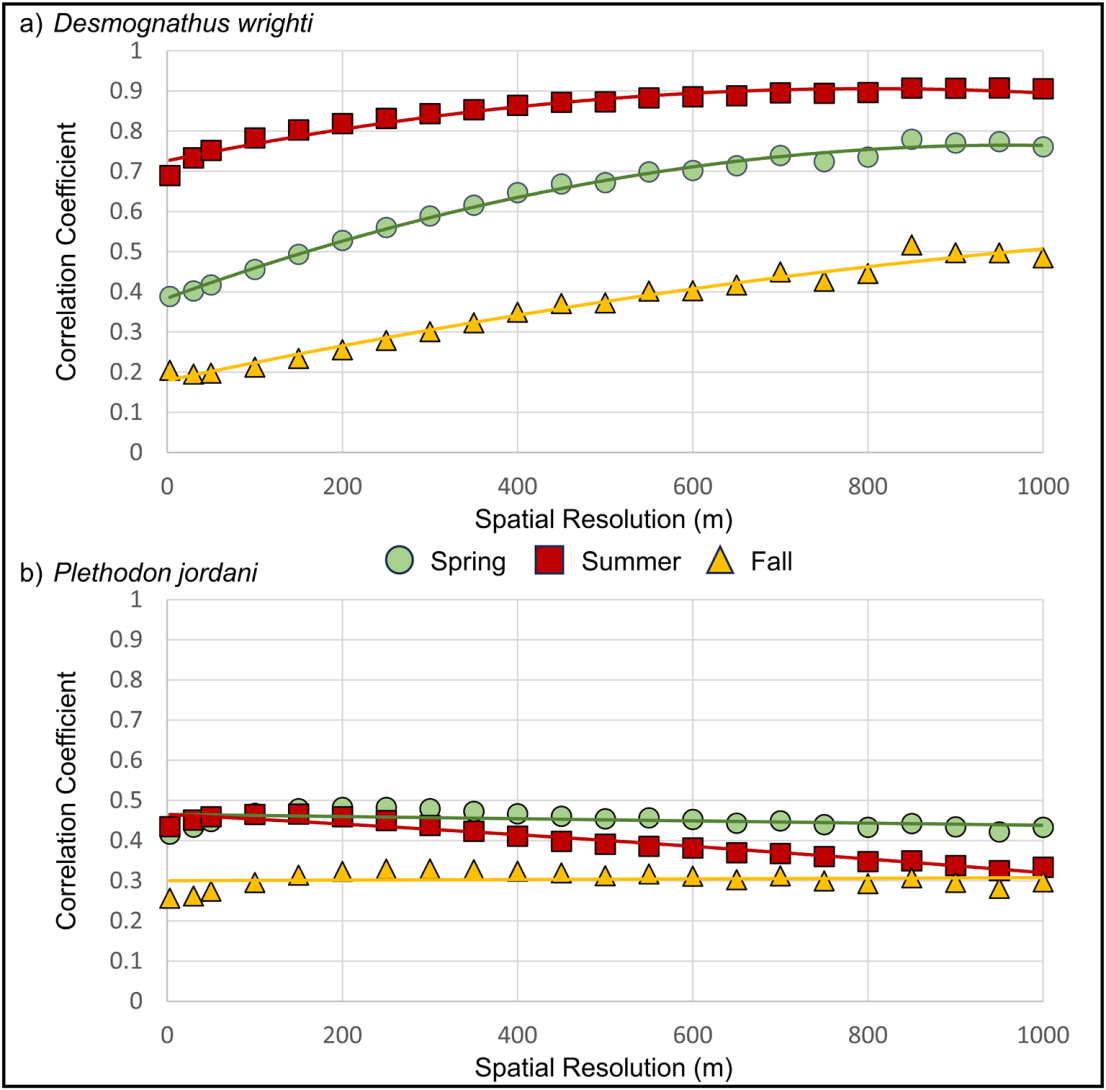
Correlation (Spearman's *ρ*) between models of microclimate species distribution models and microclimate mechanistic models at increasing levels of spatial resolution (3 to 1000 m) for (a) *Desmognathus wrighti* and (b) *Plethdon jordani*. The average correlation coefficients (Spearman's *ρ*) from each time period (2010, 2030, and 2050) are displayed for spring (circles), summer (squares), and fall (triangles). All correlations were negative, indicating low to moderate levels of agreement, but absolute values were used for graphical display.

Across a gradient of spatial resolutions (3–1000 m), we found that the agreement between SDMs and mechanistic models increased at spatial resolutions coarser than our 3 m models, but model agreement plateaued or slightly declined at different spatial resolutions for each species, season, and year, indicating different levels of spatio‐temporal model convergence (Figure 4). For *D. wrighti* , correlation coefficients typically increased at coarser spatial resolutions, but correlation coefficients began to plateau at 400–800 m spatial resolution, depending on year and season (Figure 4, Figure S8). For *P. jordani* , correlation coefficients were lower on average overall, and they plateaued or began declining at 200–400 m spatial resolution, depending on year and season (Figure 4, Figure S8).

### 3.4 Activity Corridors

**FIGURE 5:**
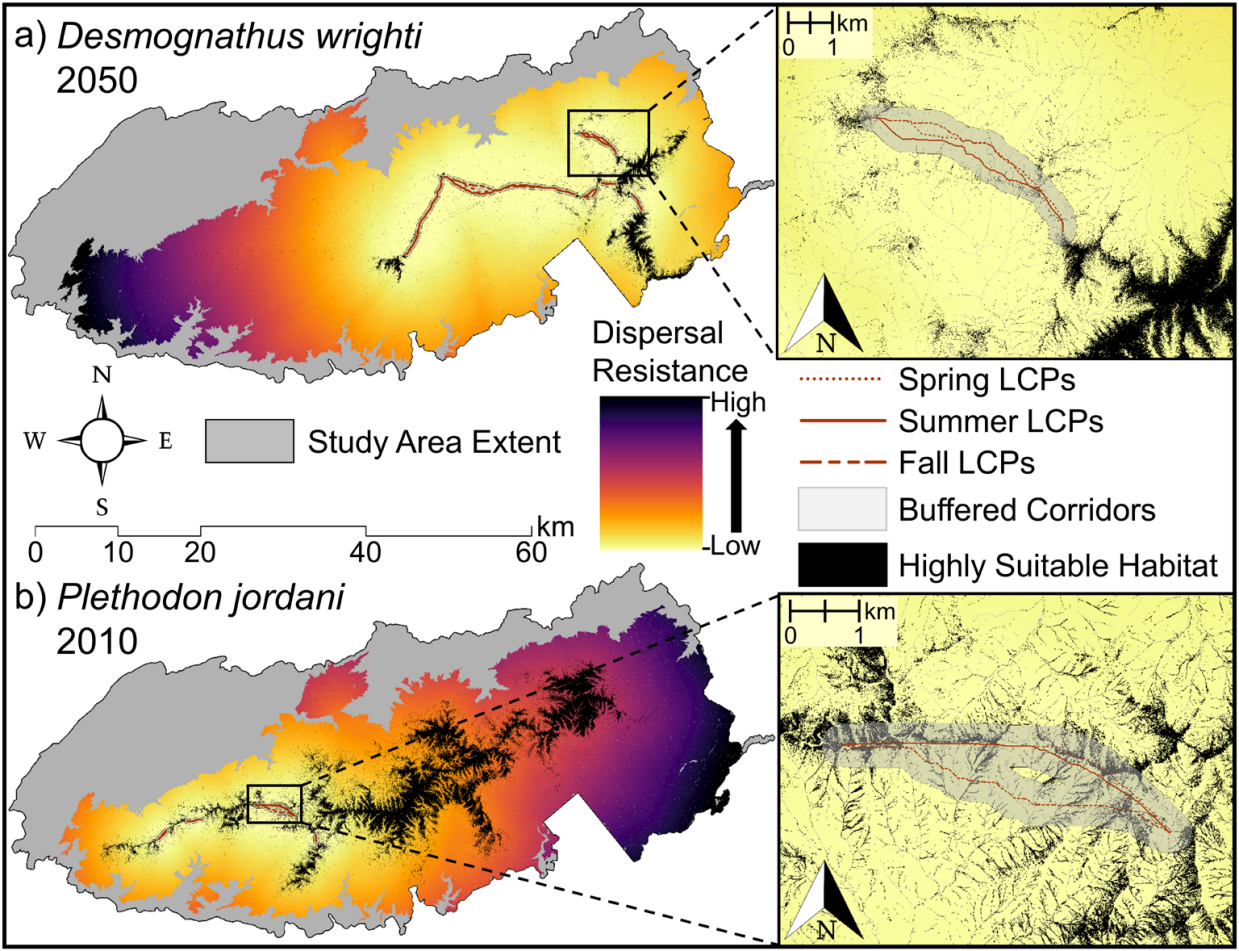
Least cost pathways (LCP) and buffered corridors of salamander surface activity between contiguous areas (≥ 1 km^2^) of highly suitable microclimate habitat from species distribution models (black) for (a) *Desmognathus wrighti* in 2050 and (b) *Plethodon jordani* in 2010. LCPs are layered on top of the resistance layers built for least‐cost path modeling. Inset maps show zoomed in areas to demonstrate that LCPs vary among seasons.

Least‐cost path analysis revealed that projected pathways of activity between areas of highly suitable habitat lengthened considerably under 2050 microclimate conditions, with an average increase in length between suitable habitat patches of 4.2 km for *D. wrighti* and 2.4 km for *P. jordani* (Table 1, Figure 5, Table S5, Figures S9–S11). Projected activity pathways also varied seasonally. Seasonal variation among LCPs was great enough to cause activity corridors (i.e., 300 m buffered LCPs) to diverge in some locations, indicating that potential corridor routes could differ significantly over time (Figure 5). The fragmentation metrics calculated within activity corridors increased substantially for both species by 2050 (Table 1). For *D. wrighti* , Euclidean nearest‐neighbor distance was projected to increase by 34.0%, patch density by 46.9%, and perimeter‐to‐area ratio by 23.8% between 2010 and 2050, on average (Table 1). For *P. jordani* , nearest‐neighbor distance was projected to increase by 19.8%, patch density by 45.1%, and perimeter‐to‐area ratio by 19.4% by 2050, on average (Table 1).

## 4 Discussion

We found poor to moderate agreement between correlative SDMs and mechanistic models that were developed with fine‐resolution, microclimate data. However, model agreement varied depending upon temporal period and spatial resolution (Table 1, Figures 3, 4, 5, Figure S8). Understanding these spatio‐temporal mismatches in model agreement is important as we continue to search for robust approaches to integrate microclimate‐derived predictions from correlative SDMs and mechanistic models at biologically appropriate spatio‐temporal resolutions. This is an essential step for accurately predicting species distributions under changing environmental conditions (Hijmans and Graham 2006; Kearney and Porter 2009; Lenoir et al. 2017; Lembrechts et al. 2018) and reducing uncertainty for land managers tasked with developing and implementing conservation and management plans (Schuwirth et al. 2019); especially when considering smaller species that are more vulnerable to climate change (Cushman 2006; Hoegh‐Guldberg et al. 2008; Clusella‐Trullas et al. 2011). Our analyses highlight the potential challenges for integrating fine‐resolution models for species reliant on microclimate conditions at local scales and raise a variety of spatio‐temporal issues.

Spatially, we found increased model agreement across broader geographical extents and at coarser spatial resolutions than our 3 m models (Figures 3 and 4, Figures S6 and S8). For example, we found low to moderate model agreement across the elevational range of both our study species, but model agreement was noticeably weaker when we restricted the comparison to areas where SDMs predicted highly suitable microclimate habitat (Figure 3, Figure S6). The plethodontid salamanders focused on in this study are high elevation specialists (Dodd 2004; Peterman et al. 2016; Hocking et al. 2021), and our SDMs indicated that high elevation areas are highly suitable habitat. Therefore, the stronger model agreement we observed across the full extent of GSMNP is likely due to the increased variation in elevation across the mountainous terrain of the park and the significant effect that elevation, and other topographic variables, have on climate and climate‐derived variables, such as the bioclimatic variables, SMR, and VPD models used in this study. However, we also found that model agreement initially increases with decreasing spatial resolution but eventually converges at medium to coarse resolutions (Figure 4, Figure S8). This indicates an important contradiction in fine‐resolution, microclimate modeling of species' distributions in which we may have a need to integrate microclimate data at biologically meaningful spatio‐temporal resolutions for certain species or studies, but the variation that occurs at the very fine spatial scales presented in this study may result in weakened model agreement between correlative and mechanistic approaches. Reconciling discrepancies among model predictions at such fine spatial resolutions therefore represents an important direction for future research.

Previous studies suggest that mechanistic models may poorly predict a species' range because they tend to account for only a limited set of abiotic constraints, overlooking other abiotic or biotic factors that may limit distributions (Buckley et al. 2010). Additionally, it has been long understood that measurements and statistics are sensitive to spatial scale, and that coarse‐resolution models may average out the variation in fine‐resolution models (Wiens 1989), resulting in stronger bivariate relationships, such as correlation coefficients at coarser resolutions (Lam and Quattrochi 1992; Quattrochi and Goodchild 1997; Perveen and James 2009, 2011). As such, it is unsurprising that we found stronger agreement among our microclimate SDM and mechanistic models at coarser spatial resolutions than at fine resolutions; however, this may be an important, yet potentially neglected, issue as fine‐resolution microclimate data become increasingly available and used to develop SDMs and mechanistic models. As more advanced, fine‐resolution models become easier to develop, researchers may need to balance the benefits of spatial resolution, study area extent, and the importance of representing different biological processes that constrain geographic ranges. For certain species or studies, fine‐resolution modeling approaches may be beneficial, but for other species or research questions, moderate or coarser spatial resolutions may be better suited and provide the potential benefit of more fully accounting for the variety of factors that constrain species' ranges. It may also be useful to consider multi‐scale approaches for assessing the appropriate resolution or using multiple resolutions for targeting conservation efforts (see Macdonald et al. 2019; Poor et al. 2020). For example, a multi‐step process could be employed whereby coarse‐resolution SDMs are used to capture species' ranges at a broader climatic level, and fine‐resolution microclimate models, such as hybrid SDMs or mechanistic approaches, are used to target specific microhabitats or species' functionality at the local scale. Researchers could also go beyond statistically summarizing the mean of fine‐scale values within the coarse‐resolution grids and leverage the entire distribution of values to generate other biologically relevant estimates (e.g., median values, spread). These types of multi‐scaled spatial prioritization approaches could be beneficial for targeting areas of conservation with the efficiency, flexibility, and alleviation of risk that conservation managers prefer (Possingham et al. 2006).

Although spatial resolution is an important consideration for correlative and mechanistic models, our results highlight temporal misalignments as well. Most notably, our model comparisons indicated much higher levels of model agreement between microclimate SDMs and mechanistic models during summer months, compared to spring and fall, and our LCP models demonstrated the potential for significant variation in potential activity corridors depending on season (Table 1, Figures 3 and 5, Figures S9–S11). This finding may indicate that the dominant constraints vary seasonally. For example, in summer, temperature may strongly constrain the surface activity of plethodontid salamanders (Petranka 1998; Dodd 2004; Connette et al. 2015; Gade et al. 2020), but biotic interactions could be the more dominant factor in spring or fall when temperature is less constraining. Ultimately, temporally misaligned models may have significant impacts on management decisions, and researchers may need to consider whether mechanistic models represent the key processes limiting activity and distributions at different temporal periods.

We also found that model agreement converged at differing spatial resolutions between our two study species, ranging between roughly 200–800 m depending on the species, season, and year (Figure 4, Figure S8). Previous studies have indicated the potential for finding similar distributional predictions among correlative SDMs, mechanistic models, or hybrid approaches (see Newman et al. 2022; Lewandrowski et al. 2024), and the spatial resolutions of these models were at levels similar to the coarser resolutions in which we found model convergence. However, our results indicate that models diverge at very fine resolutions with variation on the spatial level of model convergence, suggesting that the different model approaches capture scale‐dependent processes that are averaged out at coarser resolutions. As the availability of fine‐resolution microclimate data increases, it will be important to continue to assess whether microclimate SDMs and mechanistic approaches show this type of divergence among different species and within different environments. Previous studies have shown that fine‐resolution microclimate SDMs may be useful in quantifying species' ranges more accurately (Stark and Fridley 2022; Stickley and Fraterrigo 2023; Haesen et al. 2023). Fine‐resolution microclimate models may also provide robust inputs for assessing an array of occupancy dynamics at the population or individual scale, such as population demographics, metapopulation dynamics, and movement of individuals (Hortal et al. 2010). However, in many cases, conservation managers may not have the need for these specific types of modeling approaches or models developed at such fine spatial resolutions. Instead, microclimate models developed at finer temporal resolutions using integrated approaches may help increase confidence for better assessing biogeographic processes (Hortal et al. 2010).

For salamanders, our microclimate‐derived projections under future climate conditions indicated a significant loss of suitable microclimate habitat and considerable increases in habitat fragmentation within activity corridors (Table 1, Figure 2, Table S5). However, the estimated loss of highly suitable habitat is moderate compared to previous plethodontid salamander SDMs that used coarse‐resolution, free‐air temperature inputs (see Stickley and Fraterrigo 2023). This finding indicates the potential for using fine‐resolution microclimate data for assessing species' distributions or re‐distributions under changing climate conditions for the variety of species reliant on microclimatic regimes (Stark and Fridley 2022; Stickley and Fraterrigo 2023; Haesen et al. 2023); however, there were limitations to the present study that must be acknowledged. For example, we compared models that use different input variables, such as bioclimatic variables for SDMs and direct microclimate temperature estimates for the SMR and VPD models. This was done because bioclimatic variables have been shown to be physiologically important for species distributions (Title and Bemmels 2018) and better for representing physiological limitations based on seasonal trends (O'Donnell and Ignizio 2012). However, every model we developed in this study (current and future bioclimatic SDMs, SMRs, and VPDs) used the identical microclimate temperature dataset as the initial input variable. Therefore, we believe the comparisons among models are valid, but the findings should be considered conservative, and more studies should assess model agreement between SDMs and other mechanistic modeling approaches across a wider range of organisms to evaluate the relevant spatio‐temporal scales. Furthermore, our approach for integrating the SMR and VPD layers was a basic attempt to locate areas in GSMNP that potentially contained both lower energetic costs of maintenance and increased surface activity, but more advanced methods for layer integration should be considered for future projects. It should also be noted that the SMR estimates used in our mechanistic models were based on statistically significant linear models of body mass, but the proportion of variance explained by the body mass models was moderate (see Supporting Information). The SMR model for *D. wrighti* was also based on formulas for all temperate plethodontid species (Feder 1976, 1983), and our SMR models do not account for the potential physiological plasticity and resistance to hotter periods that some salamanders may demonstrate (Riddell et al. 2018). Therefore, estimates from our final mechanistic models may not account for these important physiological aspects. More work is needed to accurately predict physiological responses to climate change for salamanders, including models that better account for body mass clines as we attempted in this work (Gifford 2016; Peterman et al. 2016). Lastly, we used optimal least‐cost path modeling between core areas to assess potential activity corridors, a relatively simplistic approach that may not account for more advanced aspects of functional connectivity modeling.

Nevertheless, our study indicates a potential contradiction in which we may need to use microclimate data at finer, more biologically meaningful resolutions to effectively model species distributions, but agreement with mechanistic approaches may be weakened at such spatio‐temporal resolutions, an issue that may need consideration to advance the development of microclimate‐derived SDMs and mechanistic models. Herein, we are limited to model comparisons across two species of contrasting body sizes in GSMNP, so there is a need to investigate the magnitude of model mismatch for more species reliant on varied microclimatic regimes. To provide accurate predictions of species' distributions and re‐distributions under altered environmental conditions, the integration of multiple model types (e.g., correlative, mechanistic, and agent‐based models) at appropriate biological scales will be important (Kearney and Porter 2009; Evans et al. 2015). The findings from this study indicate some potential spatio‐temporal issues in integrating fine‐scale, microclimate SDMs with other mechanistic approaches, but future research on this subject may help identify a balance between spatial resolution, temporal resolution, and the integration of multiple model approaches. Understanding these issues more acutely as more microclimate data become available may benefit land managers and conservation practitioners in need of understanding where species are and where they may be going.

## Author Contributions

**Samuel FitzSimons Stickley:** conceptualization (lead), data curation (equal), formal analysis (lead), funding acquisition (supporting), investigation (lead), methodology (lead), project administration (lead), resources (equal), software (lead), supervision (lead), validation (lead), visualization (lead), writing – original draft (lead), writing – review and editing (equal). **John A. Crawford:** conceptualization (supporting), data curation (equal), formal analysis (supporting), funding acquisition (equal), investigation (supporting), methodology (supporting), project administration (supporting), resources (equal), software (supporting), supervision (supporting), validation (supporting), visualization (supporting), writing – original draft (supporting), writing – review and editing (equal). **William E. Peterman:** conceptualization (supporting), data curation (equal), formal analysis (supporting), funding acquisition (equal), investigation (supporting), methodology (supporting), project administration (supporting), resources (equal), software (supporting), supervision (supporting), validation (supporting), visualization (supporting), writing – original draft (supporting), writing – review and editing (equal). **Jennifer M. Fraterrigo:** conceptualization (lead), data curation (equal), formal analysis (supporting), funding acquisition (equal), investigation (supporting), methodology (supporting), project administration (lead), resources (equal), software (supporting), supervision (lead), validation (supporting), visualization (supporting), writing – original draft (supporting), writing – review and editing (equal).

## Conflicts of Interest

The authors declare no conflicts of interest.

## Supporting information


Appendix S1.

## Data Availability

The dataset supporting the findings from this study is openly available in the Illinois Data Bank at https://doi.org/10.13012/B2IDB‐1162151_V1.
