# Supplementary material for: Spatio‐Temporal Comparisons Between Microclimate Species Distribution Models and Mechanistic Models of Potential Surface Activity
Source: Ecol Evol. 2025 Jul 16;15(7):e71813. doi: 10.1002/ece3.71813 (PMC12266774; doi:10.1002/ece3.71813)
Supplement: Supplementary file 1 — Appendix S1. [file ECE3-15-e71813-s001.docx]

**Supplementary Material**

*Microclimate Data*

All of the models in this work used the microclimate dataset from Stickley and Fraterrigo (2021). The microclimate predictions from this dataset were produced with data collected by Fridley (2009) via a network of 159 HOBO and iButton loggers. These dataloggers were placed 1 m above the forest floor, they were shielded with radiation caps, and they were attached to the north side of trees to capture the sub-canopy conditions while minimizing radiation forcing. However, it should be noted that the microclimate estimates in this work capture sub-canopy, air temperatures near the forest floor but not the exact surface or soil temperatures that salamanders experience during surface activity or burrowing. For specific datalogger details see Fridley (2009) and for microclimate modeling details see Stickley and Fraterrigo (2021).

*Microclimate Species Distribution Models*

For future temperature predictions used in SDMs, we calculated differentials between the current (2010) and future (2030 and 2050) time periods for the NASA NEX-DCP30 dataset. We also downloaded current (2010) and future (2030 and 2050) monthly precipitation maps from NASA NEX-DCP30 (Thrasher et al. 2013) and resampled these outputs to match the grid size of our microclimate dataset. We used bilinear resampling to match the NASA NEX-DCP30 climate layers with the 3 m spatial resolution of the microclimate layers.

The future temperature predictions were produced by calculating temperature differentials from resampled monthly temperature predictions for the years 2010 (2006-2010 average), 2030 and 2050 via the National Aeronautics and Space Administration Earth Exchange Downscaled Climate Projections dataset (Thrasher et al. 2013; NASA NEX-DCP30). Temperature differentials were then summed with the 2010 microclimate temperature dataset to predict 2030 and 2050 microclimate temperatures. Precipitation estimates from NASA NEX-DCP30 were also downloaded and resampled to use for precipitation inputs to SDMs (Stickley and Fraterrigo 2023).

To parameterize the MaxEnt models, we used the ‘SDMtune’ package (Vignali et al. 2022). For *Desmognathus wrighti*, a regularization multiplier of 2.0 and the inclusion of linear, product, quadratic, hinge, and threshold features produced the optimal model. For *Plethodon Jordani*, a regularization multiplier of 2.0 and the inclusion of linear, product, quadratic, and hinge features produced the optimal model.

For each species (*D. wrighti* and *P. jordani*) and time period (2010, 2030, 2050), we developed SDMs from each of the three algorithms (MaxEnt, ANN, GBM), then created a final ensemble model. To calculate the ensemble model, we ranked the MaxEnt model as our top model because MaxEnt (Elith et al. 2006) has historically been the most widely-used program with presence-only occurrence data (Elith et al. 2011). We also chose this as our top model due to the fine-tuning of model parameters.

We then ranked the models with MaxEnt being ranked first due to the model parameterization and wide-use of MaxEnt programming for presence-only modeling (Elith et al. 2011). The remaining two models were ranked based on their TSS values, which resulted in the ANN and GBM models ranked second and third, respectively. Using a simple rank order determine of weights, the ensemble models were calculated for each species and time period with our MaxEnt output as 0.5 weight, ANN as 0.33 weight, and GBM as 0.17 weight.

*Microclimate Mechanistic Models*

Plethodontid Body Mass and Elevation

To develop predictive relationships between plethodontid body mass and elevation, we used body mass measurements for each plethodontid species from Peterman et al. (2016). Peterman et al. (2016) conducted surveys in July 2012 at night and within one day of rain events. They used visual encounters to sample surface-active plethodontids and measured body mass at site locations ranging from 669-2,019 m in elevation. We fit multiple linear regression models to salamander body mass (separately for males, females, and juveniles) as a function of elevation and used a square root transformation of the response variable for the juvenile *D. wrighti* models to meet the assumptions of linear regression. For *P. jordani*, we included a cubic polynomial term to improve model fit for body mass predictions. We evaluated models using 10-fold cross validation and assessed predictive accuracy by calculating mean absolute error and root-mean-square error. Model estimates for each study species were used to predict body mass for each sex (male or female) and stage class (juvenile or adult) of the study species across a 3 m DEM raster (Stickley 2021). The final body mass models were statistically significant, but the proportion of variance explained by the body mass models varied and were moderate ranging from 0.40 r-squared to 0.55 r-squared depending on sex and stage class (Stickley 2021).

Standard Metabolic Rate

We modeled current and future SMR (volume of oxygen consumption, VO2 μl g-1 hr-1) for both study species using microclimate temperature estimates from Stickley and Fraterrigo (2021) and the body mass estimates from Stickley (2021) as described in the preceding section. We combined species specific body mass predictions that account for body mass-elevation relationships with seasonal maps of minimum microclimate temperature (Stickley and Fraterrigo 2021). Minimum temperature was used to predict minimum SMR (i.e., SMR during nighttime minimum temperatures). Minimum SMR was calculated for each sex and stage class of the study species separately using the equations of Feder (1976, 1983), which predict SMR for temperate plethodontid species as a function of temperature and body mass. For P. jordani, we also estimated SMR using a formula that incorporated body mass measurements to obtain more accurate estimates of percent change in SMR (Gifford and Kozak 2012) and averaged those predictions with the Feder (1976, 1983) predictions to reduce uncertainty. For this study, we averaged the juvenile, male, and female SMR outputs developed from Stickley (2021) to assess average monthly and seasonal SMR for each species. Further details on the climate modeling can be found in Stickley and Fraterrigo (2021, 2023). Further details on salamander body mass and SMR models can be found in Stickley (2021).

Vapor Pressure Deficit

To calculate VPD, we followed similar methodologies to Allen et al. (1998), using an ensemble model of specific humidity for future projections with the same RCP 8.5 emissions scenario used for our SDMs. Vapor Pressure Deficit is measured as the difference between the amount of water vapor the air is capable of holding at saturation less the actual water vapor in the air, which is calculated as

VPD_Min_ = e_sMin –_ e_aMin_

where (e_sMin_) is saturation water vapor pressure (kPa) during minimum temperatures and (e_a_) is actual water vapor pressure (kPa) during minimum temperatures. We used the microclimatic minimum temperature maps to estimate e_sMin_ as

e_sMin_ = 0.611 * exp(17.3 * T_min_ / T_min_ + 237.3)

where T_min_ is minimum temperature (^o^C). We estimate e_aMin_ as

e_aMin_ = RH * e_sMin_ / 100

where RH is relative humidity (%) and calculated as

RH = 100 * (SH * *p*) / e_sMin_ * 0.622

where SH is specific humidity (kg/kg) and *p* is atmospheric pressure. Atmospheric pressure is corrected for elevation as

*p* = *p*_sea level_ * (293-0.0065 * Elevation / 293)^5.26^

where *p*_sea level_ is atmospheric pressure at sea level (101.3 kPa) and Elevation is elevation in meters above sea level. Specific humidity was extracted from the Multivariate Adaptive Constructed Analogs (MACA) dataset (Abatzoglou and Brown 2012). We downloaded NetCDF files of monthly specific humidity estimates at a 4 km^2^ spatial resolution for the months of March through April during 2006-2010, 2030, and 2050. Monthly specific humidity estimates from 20 climate projections (Abatzoglou and Brown 2012; http://www.climatologylab.org/) were averaged into ensemble models for each month of these study years. We used projections estimated under Representative Concentration Pathway (RCP) 8.5 to follow identical methodologies as discussed above for SDMs. The 4 km^2^ rasters were resampled to a ~3m^2^ grid size to match the spatial resolution of the other raster datasets used for the VPD calculation.

*Comparing Microclimate SDMs and Mechanistic Models*

To compare model agreement, we calculated Spearman’s correlation coefficient between microclimate SDMs and mechanistic model outputs. We extracted 100,000 points across the study area due to calculations being computationally expensive with such fine-resolution data layers. We tested the extracted values for issues with spatial autocorrelation or violations of statistical assumptions. We tested for spatial autocorrelation using the Spatial Autocorrelation (Global Moran's I) tool with ESRI ArcGIS Pro®. No variables indicated spatial autocorrelation when tested across the entire study area (Moran’s I values between 0.03 – 0.05). When testing for spatial autocorrelation within areas of highly suitable habitat, one SDM variable (*P. jordani* SDM for 2030) had a Moran’s I value of 0.31, indicating low spatial autocorrelation. We tested statistical assumptions (data normality, non-independence, non-linear, etc.) for each variable of extracted point data (i.e., SDM values for probability of suitable habitat and values from mechanistic models extracted from 100,000 points) for each species and for each season (spring, summer, fall) and time period (2010, 2030, 2050). Some variables indicated non-normal distributions. The relationships between correlative SDMs and mechanistic models also demonstrated non-linear or non-independence for some time periods. Therefore, we used the Spearman’s correlation coefficient, which is a nonparametric approach to assessing correlations between variables that do not meet normality or other statistical assumptions (e.g., non-linear and dependent relationships).

*Microclimate Activity Corridors*

To assess fragmentation metrics within potential activity corridors , we calculated mean Euclidean nearest-neighbor (ENN) in which higher values indicate increased distances between habitat fragments (Hargis et al. 1998), patch density (PD) in which higher values suggest increased habitat fragmentation (McGarigal and Marks 1995), and the perimeter-to-area ratio (PA) in which higher values indicate increasingly complex habitat shapes and increased edge habitat. The ENN is the average Euclidean distance (m) between two patches of habitat. Patch Density equals the number of patches in the landscape, divided by total landscape area (m2), multiplied by 10,000 and 100 (to convert to 100 hectares). Perimeter to area ratio is the average perimeter length (m) divided by average area (m). All metrics were manually calculated using the attribute table from each variable in ESRI ArcGIS Pro® v. 3.1–3.3 (Esri 2024).

**Data Availability**

The dataset supporting these findings is openly available in the Illinois Data Bank at <https://doi.org/10.13012/B2IDB-1162151_V1>.

**Literature Cited**

Abatzoglou JT, Brown TJ (2012) A comparison of statistical downscaling methods suited for wildfire applications, International Journal of Climatology 32:772-780

Allen RG, Pereira LS, Raes D, Smith M (1998), Crop evapotranspiration—Guidelines for computing crop water requirements-FAO Irrigation and drainage paper 56, FAO, Rome, 300, 6541.

Elith J, Ferrier S, Guisan A, et al (2006) Novel methods improve prediction of species ’ distributions from occurrence data. 29:129–151

Elith J, Phillips SJ, Hastie T, Dudı M (2011) A statistical explanation of MaxEnt for. 43–57. https://doi.org/10.1111/j.1472-4642.2010.00725.x

ESRI (2024) ArcGIS Pro: Release 3. Redlands, CA: Environmental Systems Research Institute.

Feder ME (1976) Oxygen consumption and body temperature in neotropical and temperate zone lungless salamanders (Amphibia: Plethodontidae). J Comp Physiol- B 110:197–208. https://doi.org/10.1007/BF00689308

Feder ME (1983) Integrating the Ecology and Physiology of Plethodontid Salamanders Author ( s ): Martin E . Feder Published by : Allen Press on behalf of the Herpetologists ’ League Stable URL : https://www.jstor.org/stable/3892572 REFERENCES Linked references are availa. 39:291–310

Fridley JD (2009) Downscaling climate over complex terrain: high finescale (<1000 m) spatial variation of near-ground temperatures in a montane forested landscape (Great Smoky Mountains). Journal of Applied Meteorology and Climatology 48: 1033–1049.

Gifford ME, Kozak KH (2012) Islands in the sky or squeezed at the top? Ecological causes of elevational range limits in montane salamanders. Ecography (Cop) 35:193–203. <https://doi.org/10.1111/j.1600-0587.2011.06866.x>

McGarigal K and Marks BJ (1995) FRAGSTATS: spatial pattern analysis program for quantifying landscape structure. US Dep Agric For Serv, General Technical Report PNW- GTR-351.

Peterman WE, Crawford JA, Hocking DJ (2016) Effects of elevation on plethodontid salamander body size. Copeia 104:202–208. https://doi.org/10.1643/OT-14-188

Stickley SF (2021). Incorporating Microclimate into Habitat Suitability Analysis for Plethodontid Salamanders [Doctoral dissertation, University of Illinois Urbana-Champaign] https://hdl.handle.net/2142/113145

Stickley SF, Fraterrigo JM (2021) Understory vegetation contributes to microclimatic buffering of near-surface temperatures in temperate deciduous forests. Landsc Ecol 0123456789: https://doi.org/10.1007/s10980-021-01195-w

Thrasher, B., J. Xiong, W. Wang, F. Melton AM and RN (2013) Downscaled Climate Projections Suitable for Resource Management. Eos Trans 94:321. https://doi.org/10.1023/B

Vignali S, Barras AG, Arlettaz R, Braunisch V (2022) SDMtune: An R package to tune and evaluate species distribution models. Ecology and Evololution, 10(20), 11488–11506

**Supplementary Tables**

**Table S1.** Bioclimatic variables for species distribution modeling inputs. All variables were based on WorldClim bioclimatic variables but developed using microclimate temperature and NASA NEX-DCP30 variables.

|  |
| --- |
| **WorldClim Bioclim (BIO) Code and Description** |
| Annual Mean Temperature |
| Mean Diurnal Range = Mean of monthly (Max Temp - Min Temp) |
| Isothermality = (Mean Diurnal Range/Temperature Annual Range) * 100 |
| Temperature Annual Range = Max Temperature of Warmest Month - Min Temperature of Coldest Month |
| Mean Temperature of Wettest Quarter |
| Mean Temperature of Driest Quarter |
| Precipitation Seasonality (Coefficient of Variation) |
| Precipitation of Wettest Quarter |
| Precipitation of Driest Quarter |
| Precipitation of Warmest Quarter |
| Precipitation of Coldest Quarter |

**Table S2.** Species distribution models (SDM) goodness of fit. MaxEnt features and regularization values selected during model comparisons are also reported. Artificial Neural Network and Boosted Regression Trees models include the average TSS score calculated during model selection with the ‘BioMod2’ package in R statistical software. The TSS was used to rank ‘BioMod2’ developed models but the AUC value is also reported.

| **Species Distribution Models Goodness of Fit** | | | | | | |
| --- | --- | --- | --- | --- | --- | --- |
|  | ***Desmognathus wrighti*** | | | ***Plethodon***  ***jordani*** | | |
|  | **2010** | **2030** | **2050** | **2010** | **2030** | **2050** |
| Average AUC for ensemble model | 0.84 | 0.85 | 0.82 | 0.84 | 0.84 | 0.82 |
| MaxEnt (50% of model weight) AUC | 0.84 | 0.84 | 0.81 | 0.84 | 0.83 | 0.79 |
| Selected MaxEnt Features | linear , product, quadratic, hinge, threshold | | | linear , product, quadratic, hinge | | |
| Selected Maxent Regularization Value | 2.0 | | | 1.8 | | |
| Artificial Neural Network Average TSS Score for model selection | 0.52 | - | - | 0.57 | - | - |
| Artificial Neural Network (33% of model weight) AUC | 0.83 | 0.84 | 0.81 | 0.83 | 0.83 | 0.82 |
| Boosted Regression Trees Average TSS Score for model selection | 0.51 | - | - | 0.56 | - | - |
| Boosted Regression Trees (17% of model weight) AUC | 0.86 | 0.86 | 0.85 | 0.86 | 0.85 | 0.85 |

**Table S3.** Mean and standard deviation for vapor pressure deficit (VPD) and standard metabolic rate (SMR) for *Desmognathus wrighti* and *Plethodon jordani* across the study extent. Species-specific, raw values reported, not the re-scaled values used for least cost pathway modeling. Standard deviation displayed within ().

| **Vapor Pressure Deficit (kPa)** | | | | | | | |
| --- | --- | --- | --- | --- | --- | --- | --- |
|  | | **2010** | | **2030** | | **2050** | |
| **Spring** | | 0.84 (0.10) | | 0.95 (0.11) | | 1.06 (0.12) | |
| **Summer** | | 0.67 (0.11) | | 0.83 (0.12) | | 0.99 (0.14) | |
| **Fall** | | 0.61 (0.11) | | 0.72 (0.12) | | 0.84 (0.13) | |
|  | | | | | | | |
| **Standard Metabolic Rate (VO2 μl g-1 hr-1)** | | | | | | | |
|  | ***Desmognathus wrighti*** | | | ***Plethodon jordani*** | | | |
|  | **2010** | **2030** | **2050** | **2010** | **2030** | **2050** | |
| **Spring** | 22.29 (1.45) | 24.09 (1.47) | 25.89 (1.91) | 99.89 (11.69) | 107.23 (12.30) | 114.56 (13.00) | |
| **Summer** | 38.50 (4.79) | 44.79 (4.79) | 51.09 (4.78) | 211.30 (26.15) | 226.25 (27.39) | 241.19 (28.66) | |
| **Fall** | 21.45 (1.97) | 23.36 (1.94) | 25.27 (2.18) | 97.20 (12.67) | 104.64 (13.51) | 112.08 (14.46) | |

**Table S4.** Percent change in mechanistic model outputs for resistance to surface activity for *Desmognathus wrighti* and *Plethodon jordani* across the study extent. Displayed is percent change in resistance between 2010 and 2050, and the percent change of resistance between seasons (spring to summer and summer back to fall) for each time period.

|  | ***Desmognathus wrighti*** | | | ***Plethodon jordani*** | | |
| --- | --- | --- | --- | --- | --- | --- |
|  | **2010** | **2030** | **2050** | **2010** | **2030** | **2050** |
|  | **Percent Change 2010 - 2050** | | | | | |
| **Spring** | 14.3 | | | 12.9 | | |
| **Summer** | 24.8 | | | 26.5 | | |
| **Fall** | 15.6 | | | 14.4 | | |
|  | **Percent Change - Seasons** | | | | | |
|  | **2010** | **2030** | **2050** | **2010** | **2030** | **2050** |
| **Spring - Summer** | 40.9 | 45.1 | 48.2 | 52.5 | 52.4 | 59.9 |
| **Summer - Fall** | -43.7 | -47.3 | -49.9 | -56.7 | -53.4 | -60.9 |

**Table S5.** Landscape metrics for *Desmognathus wrighti* and *Plethodon jordani*. Included are Euclidean nearest neighbor (m) with standard deviation in (), patch density (patch per hectare), and perimeter-area ratio (m). Also included are the distances (km) between highly suitable habitat patches based upon least-cost path (LCP) modeling.

|  | ***Desmognathus wrighti*** | | | ***Plethodon jordani*** | | |
| --- | --- | --- | --- | --- | --- | --- |
|  | **2010** | **2030** | **2050** | **2010** | **2030** | **2050** |
|  | **Euclidean Nearest Neighbor (m)** | | | | | |
| **Spring** | 7.35  (6.99) | 7.33  (7.08) | 11.48 (15.90) | 7.07  (6.45) | 8.70 (10.86) | 8.99 (10.71) |
| **Summer** | 7.42  (7.15) | 7.31  (6.93) | 11.58 (16.13) | 7.11  (6.50) | 8.76 (11.76) | 8.88 (10.12) |
| **Fall** | 7.80  (8.48) | 7.55  (7.70) | 11.16 (15.48) | 7.39  (6.91) | 8.56 (11.17) | 9.02 (10.77) |
|  | **Patch Density (patch per hectare)** | | | | | |
| **Spring** | 41.39 | 41.73 | 77.59 | 30.69 | 39.28 | 59.74 |
| **Summer** | 42.37 | 41.17 | 79.07 | 31.21 | 39.76 | 60.12 |
| **Fall** | 40.61 | 41.43 | 77.80 | 35.52 | 37.39 | 60.98 |
|  | **Perimeter-Area Ratio (m)** | | | | | |
| **Spring** | 0.32 | 0.32 | 0.41 | 0.28 | 0.31 | 0.36 |
| **Summer** | 0.33 | 0.32 | 0.42 | 0.28 | 0.31 | 0.36 |
| **Fall** | 0.32 | 0.32 | 0.42 | 0.30 | 0.30 | 0.36 |
|  | **LCP Distance Between Highly Suitable Habitat Patches (km)** | | | | | |
| **Spring** | 2.48 | 3.20 | 6.65 | 2.35 | 1.62 | 4.79 |
| **Summer** | 2.50 | 3.23 | 6.73 | 2.36 | 1.64 | 4.74 |
| **Fall** | 2.55 | 3.37 | 6.75 | 2.36 | 1.65 | 4.85 |

**Supplementary Figures**

**
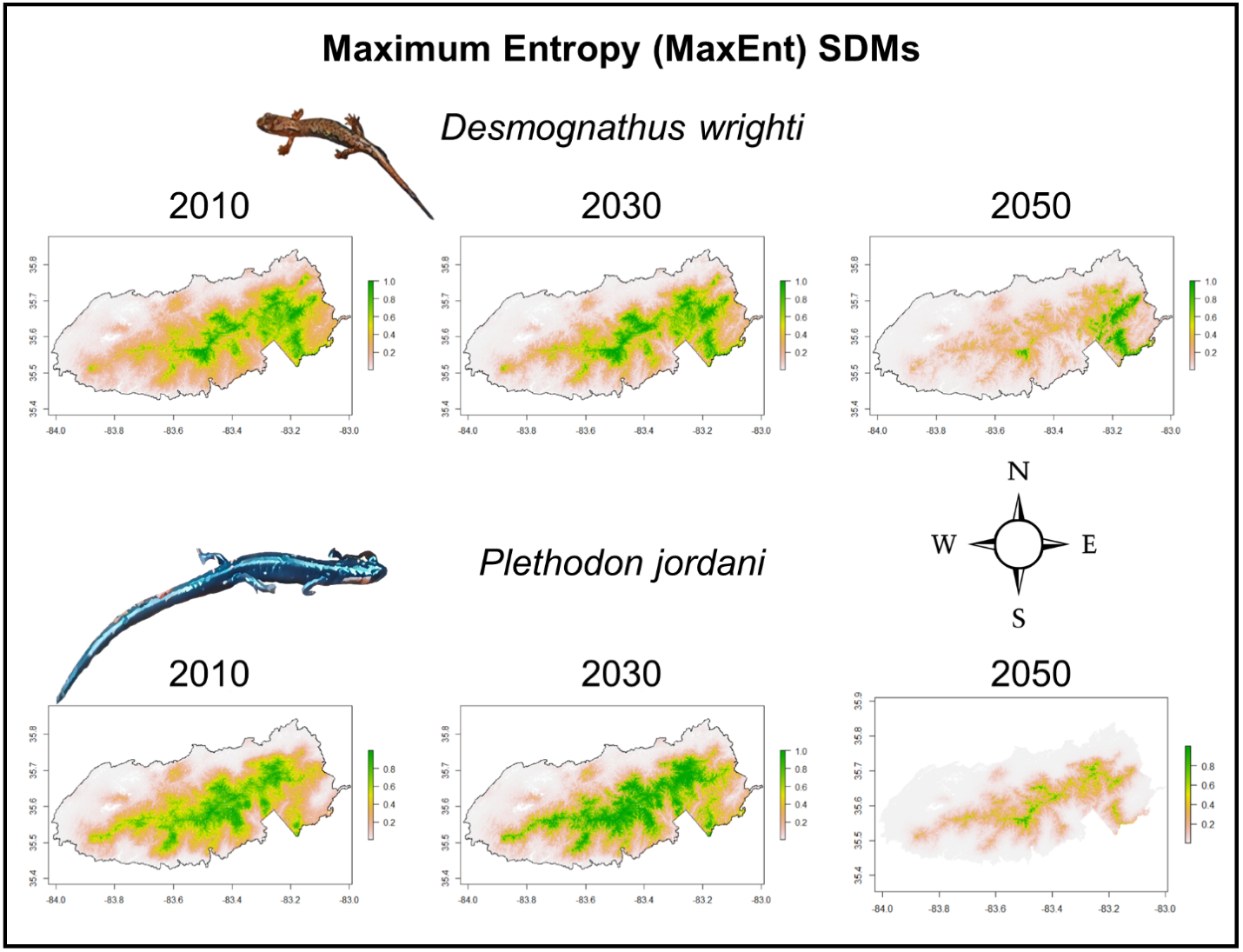
**

**Figure S1.** Maximum entropy (MaxEnt) species distribution models (SDM) for *Desmognathus wrighti* and *Plethodon jordani* for the 2010, 2030, and 2050 time periods.

**
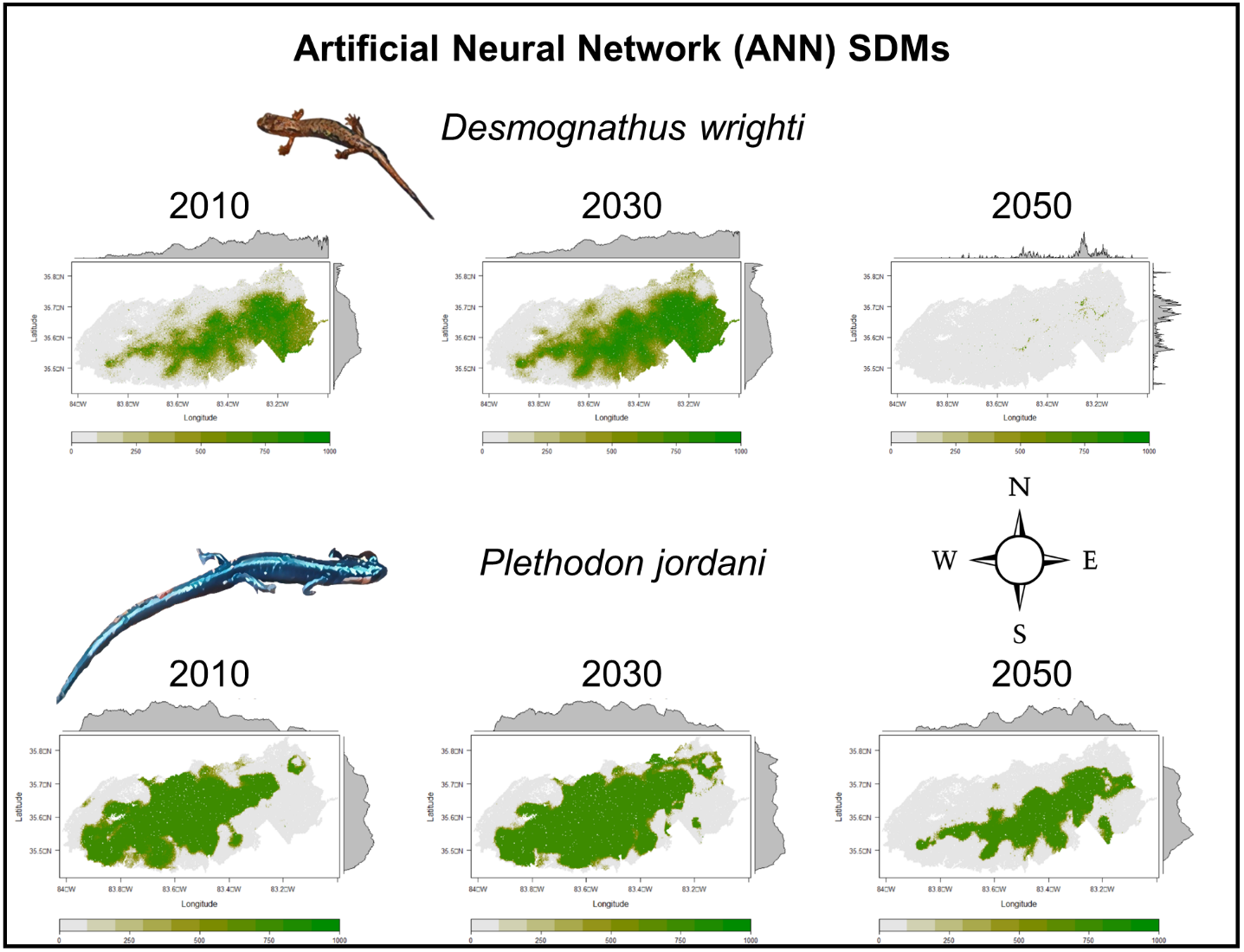
**

**Figure S2.** Artificial neural network (ANN) species distribution models (SDM) for *Desmognathus wrighti* and *Plethodon jordani* for the 2010, 2030, and 2050 time periods.

**
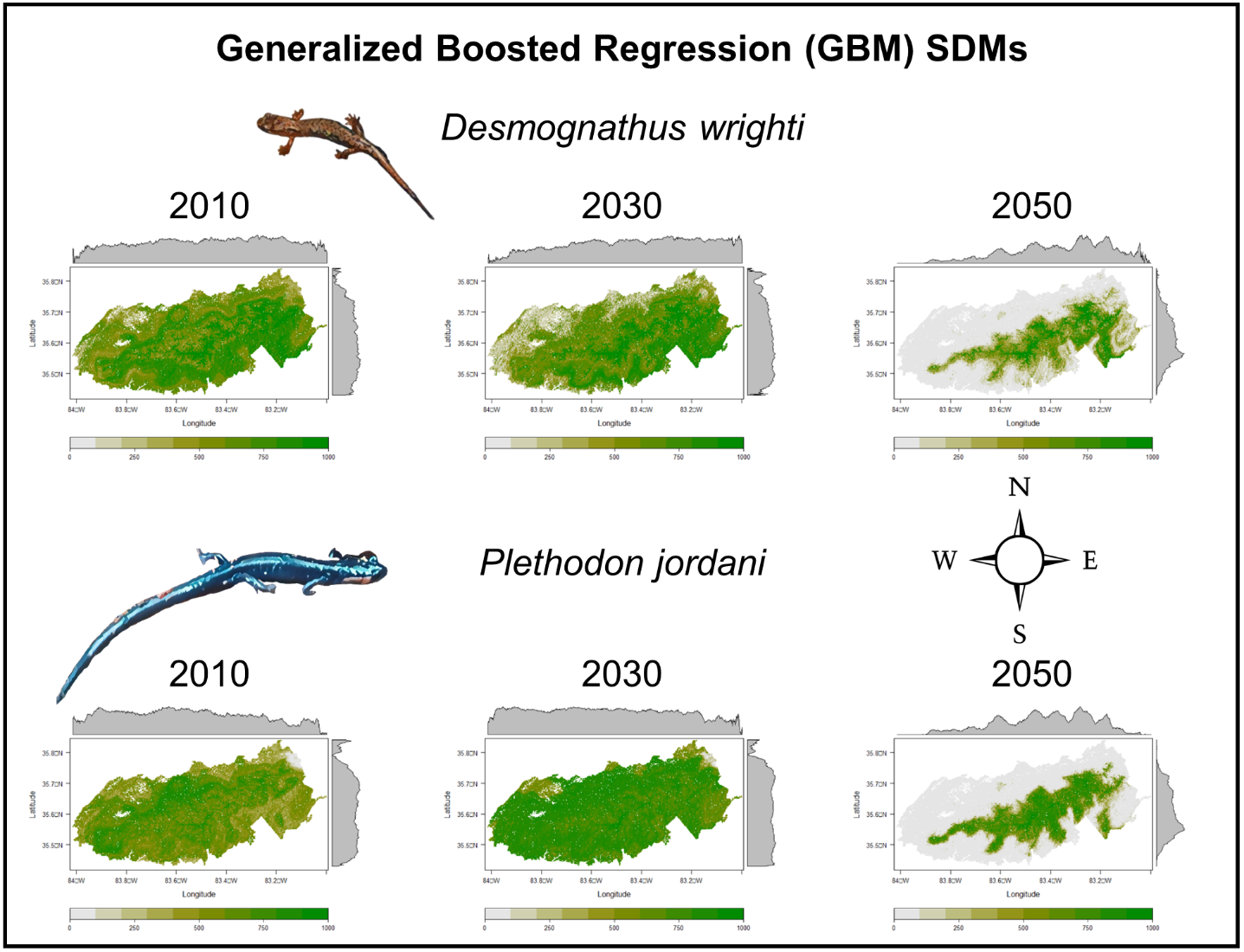
**

**Figure S3.** Generalized boosted regression (GBM) species distribution models (SDM) for *Desmognathus wrighti* and *Plethodon jordani* for the 2010, 2030, and 2050 time periods.

**
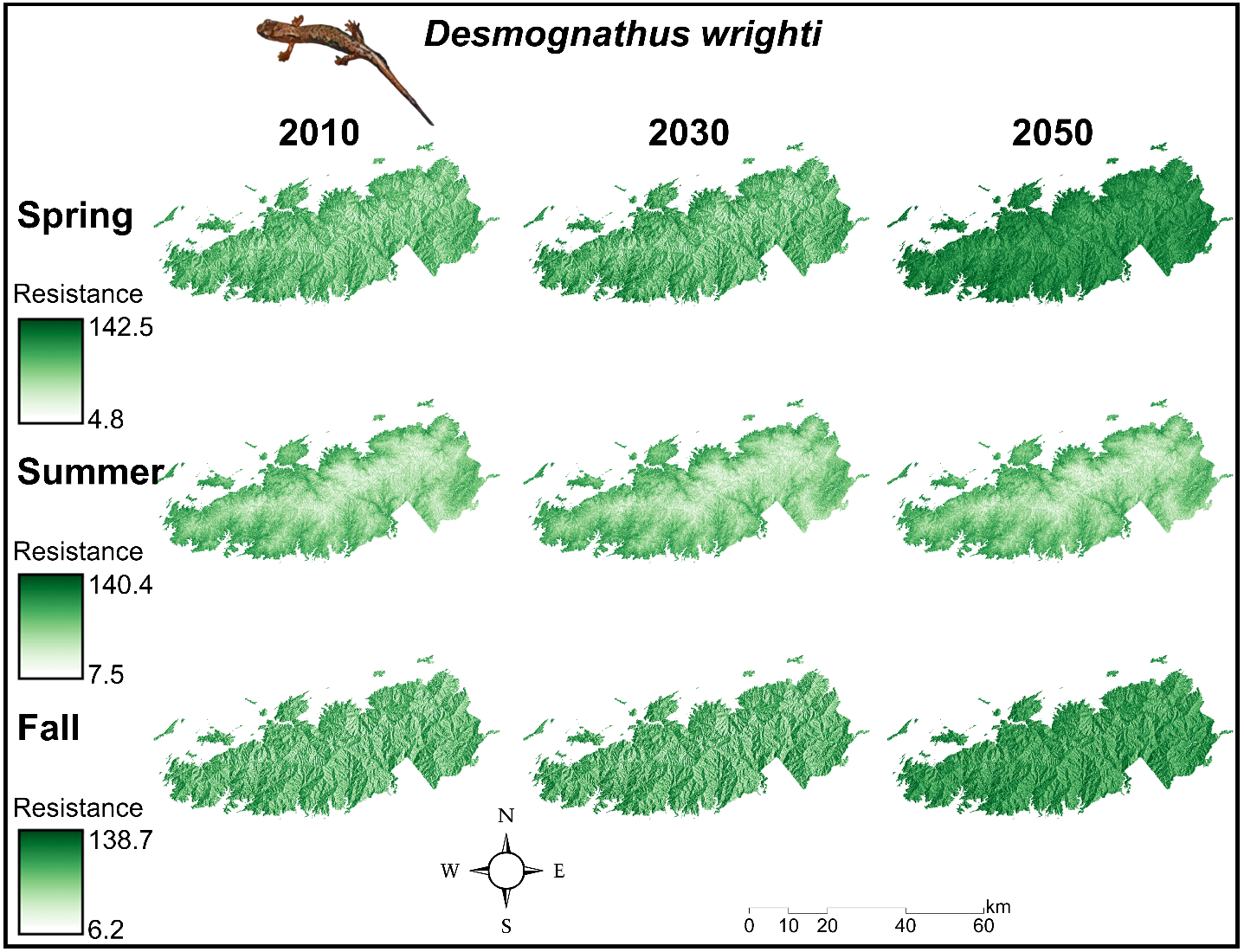
**

**Figure S4.** Mechanistic model outputs for *Desmognathus wrighti* for the spring, summer, and fall seasons of 2010, 2030, and 2050 time periods. Mechanistic odels indicate potential resistance to surface activity. Only areas within the likely elevational distribution of the study species (665 m – 2025 m) within Great Smoky Mountains National Park are included.

**
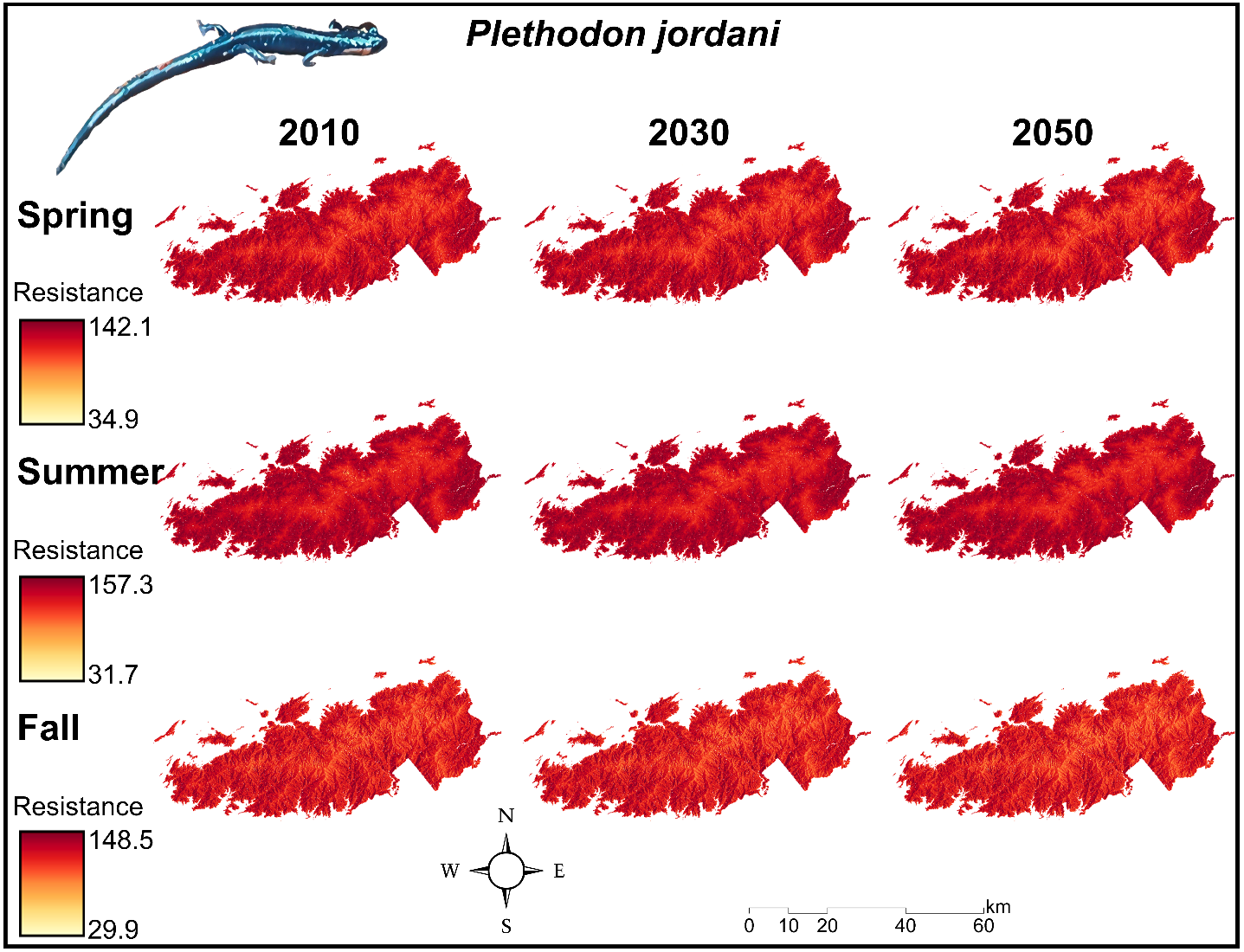
**

**Figure S5.** Mechanistic model outputs for *Plethodon jordani* for the spring, summer, and fall seasons of 2010, 2030, and 2050 time periods. Mechanistic models indicate potential resistance to surface activity. Only areas within the likely elevational distribution of the study species (665 m – 2025 m) within Great Smoky Mountains National Park are included.

**
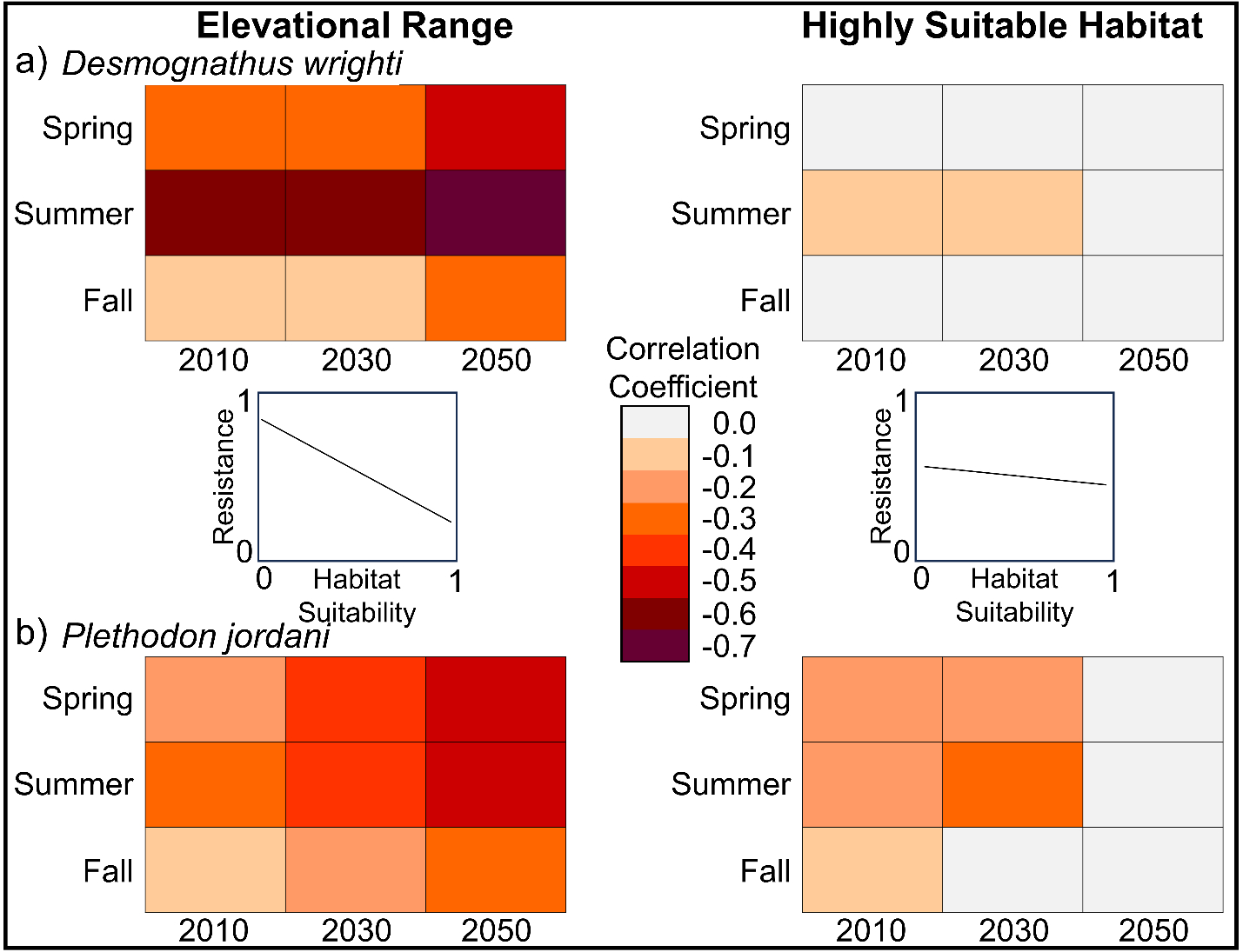
**

**Figure S6.** Spatio-temporal correlation matrices for (a) *Desmognathus wrighti* and (b) *Plethodon jordani* across the entire elevational range in Great Smoky Mountains National Park and only within highly suitable habitat (probability of suitable habitat ≥0.7). Correlation coefficients (Spearman’s ρ) indicate the level of agreement between microclimate species distribution models and resistance to surface activity from microclimate mechanistic models.


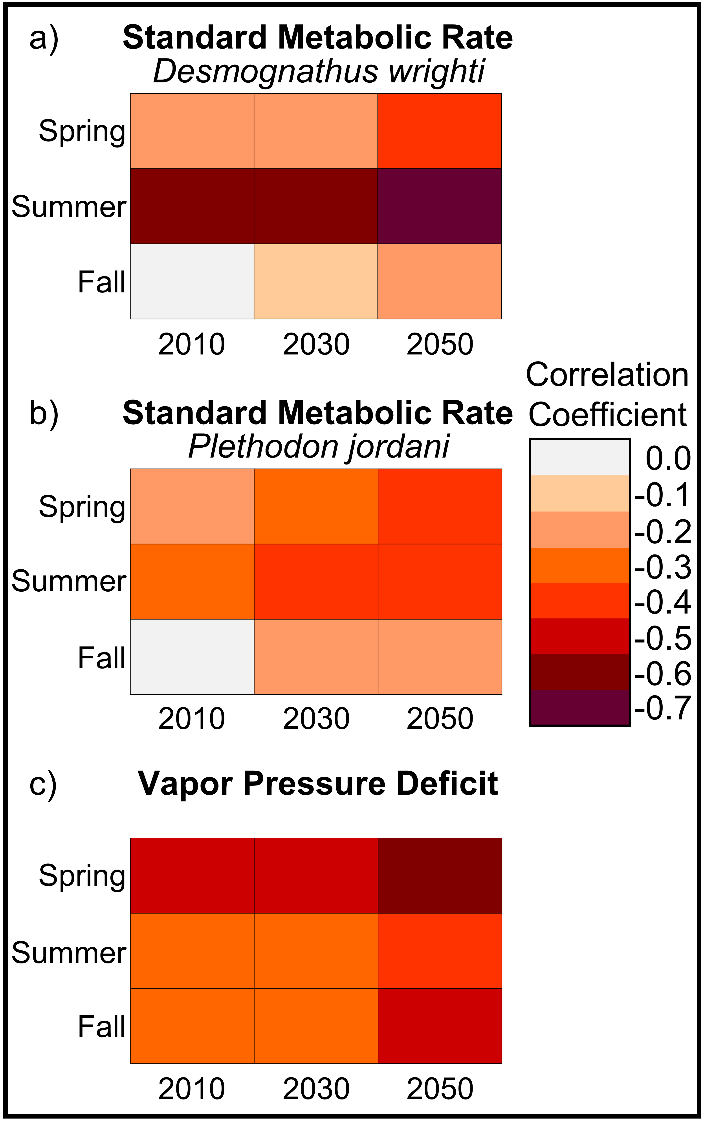


**Figure S7.** Spatio-temporal correlation matrices for (a) *Desmognathus wrighti* standard metabolic rate (VO2 μl g-1 hr-1), (b) *Plethodon jordani* standard metabolic rate (VO2 μl g-1 hr-1), and (c) vapor pressure deficit (kPa) across the entire elevational range in Great Smoky Mountains National Park. Correlation coefficients (Spearman’s ρ) indicate the level of agreement between microclimate species distribution models and either vapor pressure deficit or standard metabolic rate.


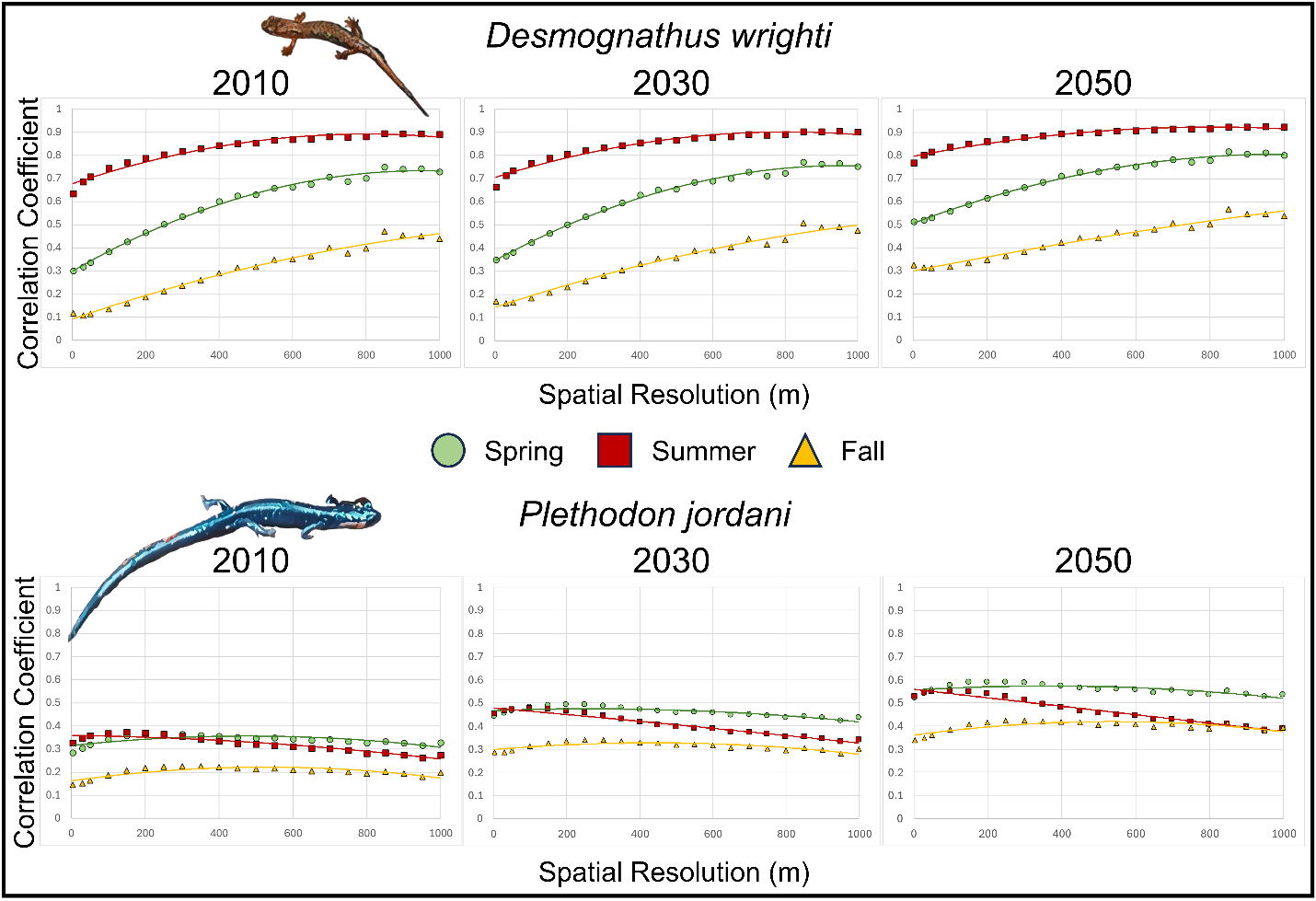


**Figure S8.** Spatio-temporal correlation matrices for (a) *Desmognathus wrighti* and (b) *Plethodon jordani* across the entire elevational range in Great Smoky Mountains National Park and only within highly suitable habitat (probability of suitable habitat ≥0.7). Correlation coefficients (Spearman’s ρ) indicate the level of agreement between microclimate species distribution models and resistance to surface activity from microclimate mechanistic models. All correlations were negative, indicating low to moderate levels of agreement, but absolute values were used for graphical display.

**
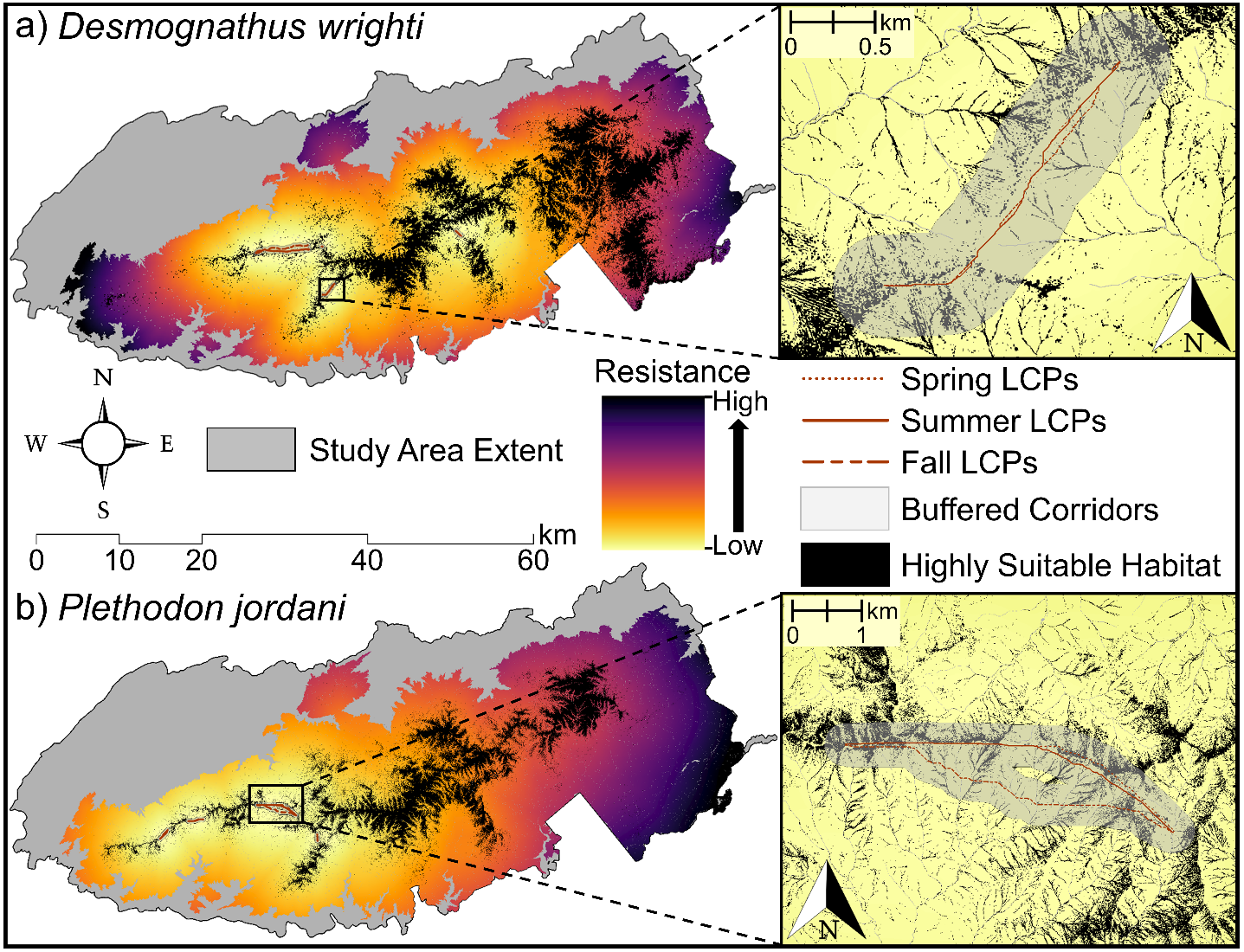
**

**Figure S9.** Least cost pathways (LCP) and buffered corridors of salamander surface activity between contiguous areas (≥ 1 km^2^) of highly suitable microclimate habitat (black) for (a**)** *Desmognathus wrighti* and (b) *Plethodon jordani* in 2010. LCPs are layered on top of the resistance layers built for least-cost path modeling. Inset maps show zoomed in areas to demonstrate that LCPs vary among seasons.

**
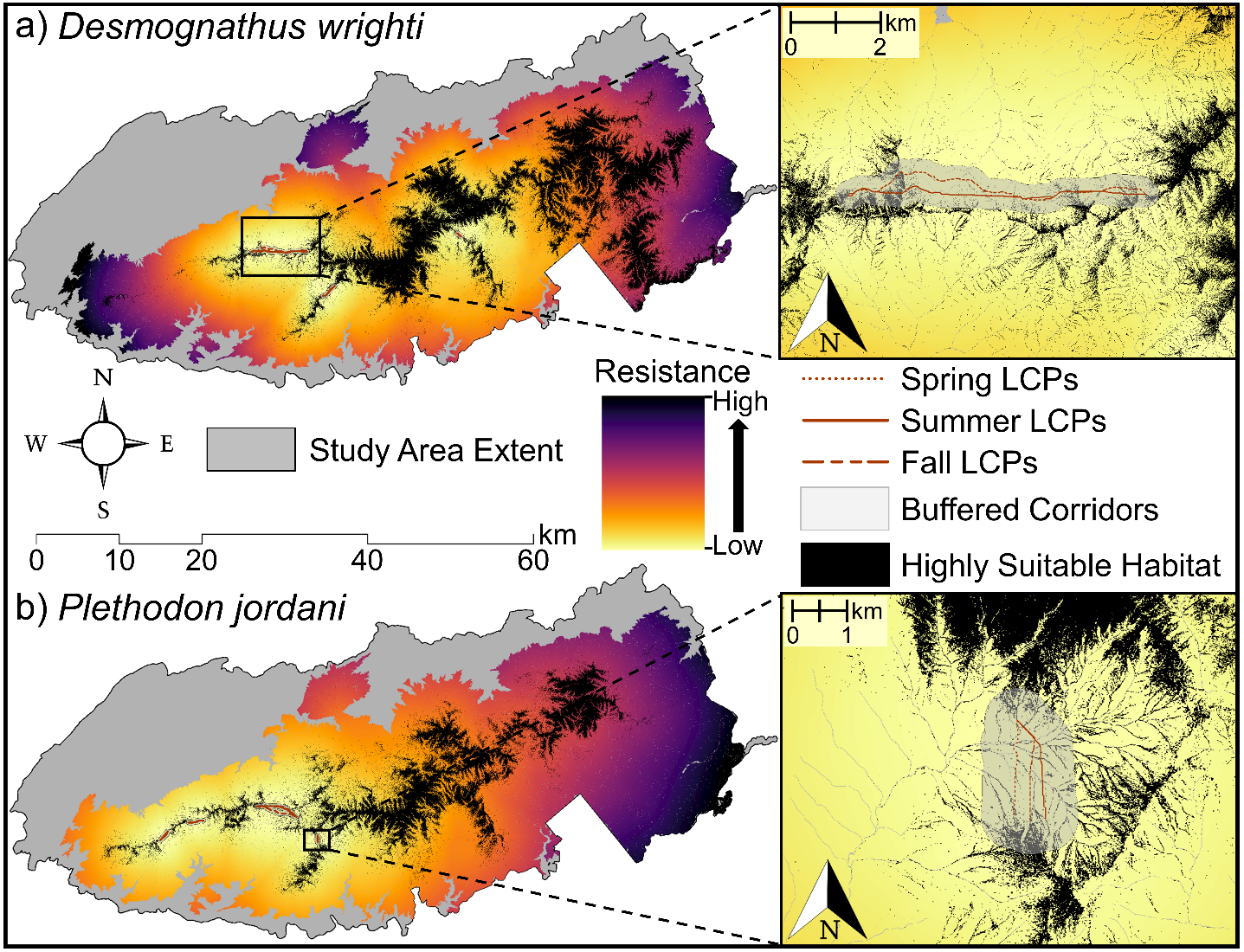
**

**Figure S10.** Least cost pathways (LCP) and buffered corridors of salamander surface activity between contiguous areas (≥ 1 km^2^) of highly suitable microclimate habitat (black) for (a**)** *Desmognathus wrighti* and (b) *Plethodon jordani* in 2030. LCPs are layered on top of the resistance layers built for least-cost path modeling. Inset maps show zoomed in areas to demonstrate that LCPs vary among seasons.

**
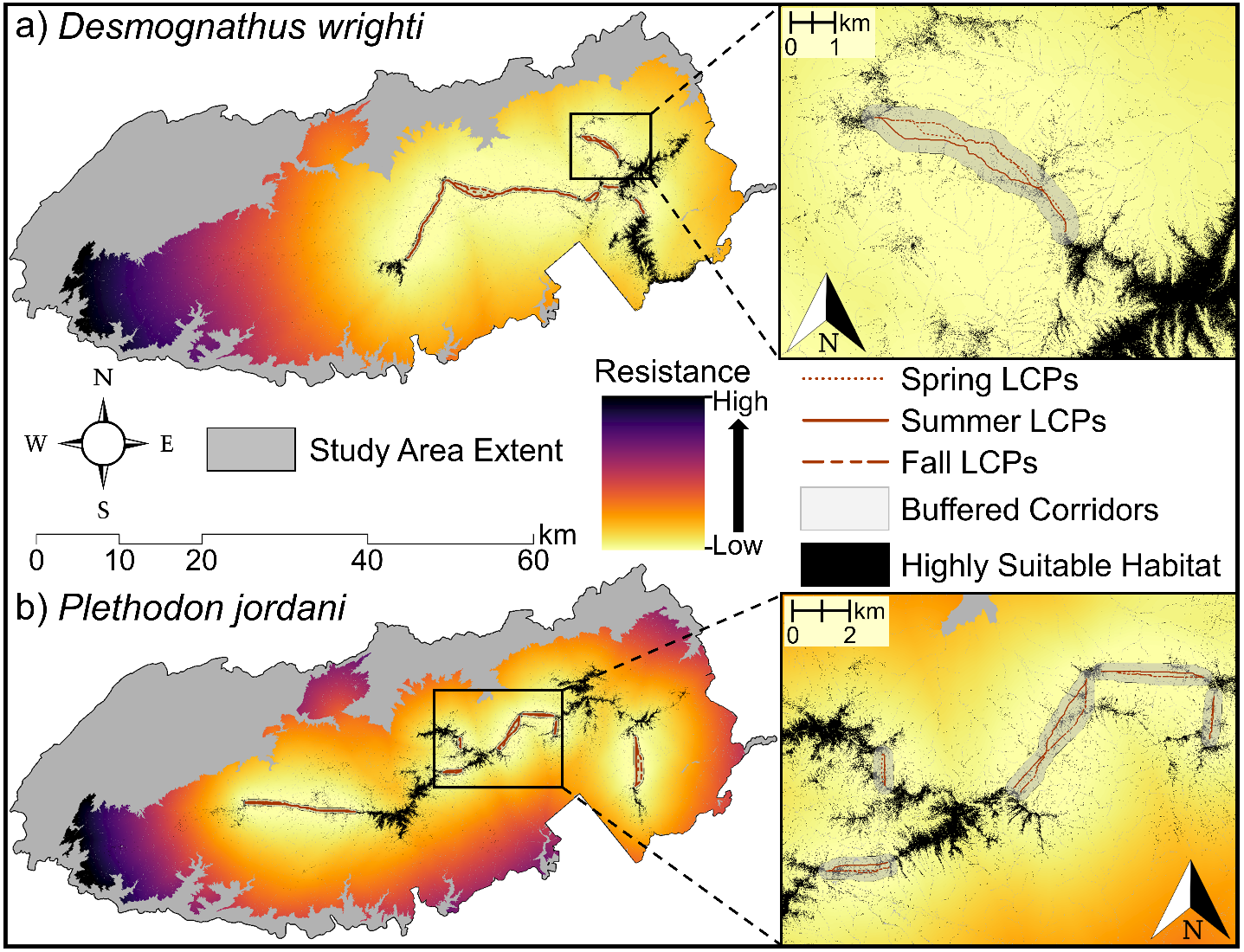
**

**Figure S11.** Least cost pathways (LCP) and buffered corridors of salamander surface activity between contiguous areas (≥ 1 km^2^) of highly suitable microclimate habitat (black) for (a**)** *Desmognathus wrighti* and (b) *Plethodon jordani* in 2050. LCPs are layered on top of the resistance layers built for least-cost path modeling. Inset maps show zoomed in areas to demonstrate that LCPs vary among seasons.
